# Combined effects of micropollutants and their degradation on prokaryotic communities at the sediment–water interface

**DOI:** 10.1038/s41598-024-67308-y

**Published:** 2024-07-22

**Authors:** Adrien Borreca, Stéphane Vuilleumier, Gwenaël Imfeld

**Affiliations:** 1 Institut Terre Et Environnement de Strasbourg, UMR 7063 CNRS, ENGEES, Université de Strasbourg, 67000 Strasbourg, France; 2 Génétique Moléculaire, Génomique, Microbiologie, UMR 7156 CNRS, Université de Strasbourg, Strasbourg, France

**Keywords:** Micropollutants, Sediment–water interface, Cocktail effect, Microcosms, Prokaryotic communities, Environmental monitoring, Microbial ecology, Water microbiology, Environmental impact

## Abstract

Pesticides and pharmaceuticals enter aquatic ecosystems as complex mixtures. Various processes govern their dissipation and effect on the sediment and surface waters. These micropollutants often show persistence and can adversely affect microorganisms even at low concentrations. We investigated the dissipation and effects on procaryotic communities of metformin (antidiabetic drug), metolachlor (agricultural herbicide), and terbutryn (herbicide in building materials). These contaminants were introduced individually or as a mixture (17.6 µM per micropollutant) into laboratory microcosms mimicking the sediment–water interface. Metformin and metolachlor completely dissipated within 70 days, whereas terbutryn persisted. Dissipation did not differ whether the micropollutants were introduced individually or as part of a mixture. Sequence analysis of 16S rRNA gene amplicons evidenced distinct responses of prokaryotic communities in both sediment and water. Prokaryotic community variations were mainly driven by matrix composition and incubation time. Micropollutant exposure played a secondary but influential role, with pronounced effects of recalcitrant metolachlor and terbutryn within the micropollutant mixture. Antagonistic and synergistic non-additive effects were identified for specific taxa across taxonomic levels in response to the micropollutant mixture. This study underscores the importance of considering the diversity of interactions between micropollutants, prokaryotic communities, and their respective environments when examining sediment–water interfaces affected by multiple contaminants.

## Introduction

As of 2020, the number of officially registered chemicals for production and application has exceeded 350,000, and is expected to continue to increase ^1^. In particular, the rise of micropollutants poses a significant challenge. Biocides, additives, and pharmaceuticals are now well-recognized as potential threats to ecosystems, biodiversity, and human health^2^. In this context, aquatic ecosystems are particularly relevant, since micropollutants infiltrate surface and groundwater systems as complex mixtures through a variety of entry points. Pharmaceuticals and personal care products are released to surface waters via wastewater treatment facilities where their transformation is frequently incomplete^3^. Pesticides employed in agriculture^4^ and building materials^5^ may also reach aquatic ecosystems directly.

The sediment–water interface (SWI) plays a pivotal, dual role as a sink and as a hotspot for dissipation and transformative processes of micropolluants^6–8^ within the hyporheic zone of continental rivers^3,9,10^. The hyporheic zone, characterised by intense exchange and reactivity between surface water and groundwater beneath the riverbed, serves as a vital detoxification organ for rivers^11^. High microbial diversity and strong exchange flows within this zone enhance the biodegradation and attenuation of organic contaminants^9,12^. These strong exchange flows ensure a continual supply of nutrients essential for microbial activity and contaminant transformation^9^. In addition, the diverse physical conditions and gradients within the hyporheic zone create varied microenvironments that support specialised microorganisms capable of degrading a wide range of contaminants^9^. In particular, heterotrophic biofilms—communities of microorganisms attached to the interface between running water and sediment—play a pivotal role in the biochemical transformation of contaminants such as pesticides^13,14^. This underscores the critical function of this interface in natural purification processes of rivers.

Micropollutant partitioning between the aqueous phase and the sediment at the SWI creates diverse exposure levels for biological compartments^15,16^ and affects microbial communities. These communities, typically dominated by bacteria, may be involved in the degradation of micropollutants^17^. Continuous exchange between sediment and the water column can enhance biodegradation and alter the composition of prokaryotic communities^18^. However, knowledge on the interactions between these biogeochemical processes and resulting changes in microbial communities remains scarce.

Micropollutants can significantly affect the diversity and composition of prokaryotic communities, either negatively or positively^19^. For instance, photosynthetic inhibitors such as quinclorac and bensulfuron-methyl may adversely affect non-photosynthetic microorganisms, e.g., through oxidative stress^19^. Similarly, high doses of insecticides like imidacloprid can reduce the richness and diversity of both archaeal and bacterial communities^20^. In this study, we examined the effect of metformin (MFN), an anti-hyperglycemic antidiabetic drug, metolachlor (MET), a widely used agricultural herbicide acting as a very-long-chain fatty acid (VLCFA) inhibitor, and terbutryn (TER), a biocide primarily used as an additive in construction materials and a photosystem II (PSII) inhibitor, on microbial communities in sediment–water microcosms. Although micropollutants and their transformation products^21^ do not directly target prokaryotic communities, we hypothesised that they may indirectly affect non-target microorganisms.

The fate of micropollutants at the SWI has been investigated in various laboratory experiments^15,22,23^, while studies on the transformation of MFN^24,25^, MET^26,27^, and TER^28,29^ have been conducted in aquatic ecosystems such as rivers and ponds. Despite the challenges of extrapolating results from the OECD 309 biodegradation test to real-world environments^30^, microcosms allow to capture the essence of phenomena under controlled conditions, although they do not fully replicate the complexity of a natural aquatic ecosystem^31^. Several studies have examined changes in microbial alpha-diversity in response to micropollutants, yet reported responses are often inconsistent. These responses can vary, resulting in increase^32^, reduction^33^, or no significant change in diversity^34,35^.

For instance, a study using sediment–water microcosms spiked with mixtures of up to 80 micropollutants found negligible effects at environmentally relevant concentrations (0.5 µg L^-1^), but more pronounced effects at higher concentrations (5 µg L^−1^)^30^.

Here, we conducted a laboratory microcosm study using river sediment to examine the dissipation of MFN, MET and TER individually and as a mixture, under biotic and abiotic conditions. Our study focuses on evaluating the response of prokaryotic communities at the SWI, specifically examining changes in their diversity and composition. Exposure to MFN, MET, and TER, individually or as a mixture, was expected to exert a selective pressure at the SWI, leading to the enrichment of specific bacterial and archaeal populations and altering overall community diversity and composition. We hypothesised that the micropollutant mixture at the SWI could induce non-additive effects on prokaryotic community composition, with synergistic or antagonistic interactions compared to individual micropollutants, and either enhanced or reduced overall impact on community composition. Our study included the evaluation of micropollutant dissipation kinetics, the formation of transformation products (TPs) under biotic and abiotic conditions, and comprehensive analysis of prokaryotic community composition in both water and sediment phases.

## Materials and methods

### Chemicals

Micropollutants, standards and their sources are listed in the Supporting Information (SI, Table S1). HPLC grade (purity: > 99.9%) dichloromethane (DCM), acetonitrile (ACN), ethyl acetate (EtOAc), methanol (MeOH), and anhydrous magnesium sulfate (reagent grade: > 97%) were purchased from Sigma–Aldrich. Primary-secondary amine-bonded silica (PSA) was purchased from Supelco.

### Experimental sediment

Sediment (top 10 cm) was collected at 10 spots chosen at random in a 10 m^2^ vegetated area of the Avenheimerbach riverbed in Alsace, France (48°39′58.08″ N, 07°35′36.92″ E) on 12 November 2020 (Fig. S1). The chosen agricultural area was adjacent to maize and beetroot plots where metolachlor is used in spring^36^. Sediment samples were collected from the riverbed using shovels and stored in pre-cleaned plastic boxes. Water depth at the collection site was approximately 50 cm. To minimize contamination, shovels and plastic boxes were sequentially cleaned with detergent, acetone, and 70% ethanol, followed by a final rinse with autoclaved water. Collected sediment samples were transported to the laboratory at ambient temperature. Superficial water was removed to prevent sediment dilution and the formation of distinct layers during subsequent storage. Sediment was then homogenised and stored at 4 °C until the first microcosm incubation experiment. No metformin, metolachlor, or terbutryn was detected in the river sediment.

### Laboratory microcosms

Microcosms were set up in 9 × 3 cm vials of 50 mL with a headspace. Vials were filled with a 1:6 sediment–water mixture containing sediment equivalent to 8 g dry weight combined with a total volume of 48 mL of water. Experiments were performed using ultrapure water (Elix® Advantage 5, Milipore).

This resulted in a concentration of total suspended solids (TSS) of 0.44 g L^-1^, in the typical range observed in river environments^37^. Oxic microcosms were fitted with PTFE caps pierced by a needle mounted with a 0.22 µm pore size filter (Millipore, France) to allow gas exchanges while preventing water loss and contamination^38^.

In total, 150 microcosms were established as five parallel experiments involving different contamination regimes under either biotic or abiotic conditions. Biotic experiments were conducted in triplicate, while abiotic experiments were conducted in duplicate, based on preliminary data indicating that duplicates adequately captured dissipation patterns (data not shown). Such streamlining of processing and analysis workload allocated more resources for the study of biodegradation and microbial communities. Water and sediment phases in abiotic microcosms were sterilized independently by autoclaving three times at 24 h intervals. Microcosms were incubated at 30 °C in the dark and subject to continuous orbital agitation at 120 rpm to ensure homogeneity. An initial one-week pre-incubation ensured stable partitioning of ions, nutrients, and particles in the microcosms^38,39^. The experimental temperature of 30 °C was chosen to enhance micropollutants biodegradation^40^, despite potential selection against psychrotrophic microorganisms^40^. Our results indicate that the chosen temperature did not decrease sorption of micropollutants^41^ (data not shown).

MFN, MET and TER were spiked at 17.6 µM each in the water phase, corresponding to concentrations of 2.3 mg L^−1^, 5.3 mg L^−1^, and 2.8 mg L^−1^, respectively. Microcosms were spiked either with a single micropollutant (‘ONE experiments’) or with a mixture of the three micropollutants (‘MIX experiment’). While these concentrations are much higher that typical environmental concentrations, they may represent acute concentration and facilitated the analysis of formed transformation products during degradation. The MFN stock solution (5 g L^−1^ in sterile milliQ water) was added directly to the microcosms. Stock solutions of MET and TER were prepared in ACN (5 g L^−1^). Aliquots (3 mL) of ACN solutions were initially mixed with 750 mL water and the obtained solutions stirred until complete ACN evaporation. Microcosms were then amended with the resulting aqueous micropollutant solutions as appropriate. Two sets of abiotic and biotic control microcosms (CTRL) were spiked with water that had undergone the same ACN exposure as the test microcosms, but without micropollutant addition. Considering the Henry coefficient of ACN and the duration of stirring for its evaporation, ACN was very likely completely evaporated. Sacrificial sampling was performed throughout, with microcosms collected on days 0, 15, 30, 50, and 70 for the analysis of primary micropollutants, their transformation products, pH, and dissolved oxygen concentrations. Hydrochemical analyses and DNA extractions for subsequent analysis of the prokaryotic community, in contrast, were performed on samples collected on day 0 and day 70 only. Hydrochemical analyses were conducted on single samples from CTRL microcosms.

### Chemical analyses

#### Biogeochemistry

Dissolved oxygen concentration was monitored in situ with non-invasive sensor spots (PreSens, Unisense) in all experiments. Total organic carbon (TOC) and dissolved organic carbon (DOC) were analyzed using a TOC analyzer (TOC-V-CPH Shimadzu, NF EN 1484). Major ions (NH_4_^+^, Na^+^, K^+^, Mg^2+^, Ca^2+^, Cl^-^, NO_3_^-^, SO_4_^2-^, PO_4_^3-^) were quantified by ion chromatography (Dionex ICS-5000, Thermo Scientific). Measurements were taken on day 0 and 70 for microcosms under biotic and abiotic conditions, and for water river from the sampling site. Water pH was also monitored routinely using pH paper.

#### Microbial activity

Changes in total microbial activity were assessed using the fluorescein diacetate (FDA) test^42^, which relies on enzymatic hydrolysis of fluorescein diacetate by esterases indicative of active cells. Briefly, 0.2 g of sediment was combined with 3.75 mL of phosphate buffer solution in a 15 mL polypropylene centrifuge tube. To this mixture, 50 µL of FDA reagent solution (1 mg mL^−1^) was added. The samples were thoroughly mixed until homogeneous and agitated at 500 rpm for 45 min. Following incubation, 3.75 mL of a chloroform:methanol (2:1, v:v) solution was added to stop FDA hydrolysis. Tubes were further incubated with 500 rpm agitation for an additional 20 min and then centrifuged for 3 min at 2000 rpm. After centrifugation, the absorbance of 200 µL triplicate samples of the aqueous phase (top layer) transferred into wells of a clear bottom 96-well plate was measured at 490 nm (Tecan M Nano + Infinite spectrophotometer).

#### Extraction and quantification of micropollutants

Micropollutants and their transformation products (TPs, Table S2) were extracted from water and sediment and analysed as described previously^4,38^. Quantification and limits of detection and quantification are provided in supporting method S1 and Table S2. Extraction yields are reported in Table S3, and matrix effects were negligible (Fig. S2).

Water samples were filtered on nitrocellulose GVS membranes (47 mm diameter, 0.22 µm pore size, Millipore). Pre-concentration of MET and TER was achieved using an AutroTrace 280 SPE system (Dionex) with SolEx C18 cartridges (1 g, Dionex)^4^. Quantification of MET and TER was performed by gas chromatography (GC, Trace 1300, Thermo Fisher Scientific) using a TG-5MS column (30 m × 0.25 mm ID, 0.25 µm film thickness) and mass spectrometry (MS, ISQ™, ThermoFisher Scientific). Pre-concentration minimizes the variability of eluate volume from the manual extraction process and ensures a consistent final volume of 1 mL in ACN. Liquid chromatography (UHPLC, Ultimate 3000, Thermo Fisher Scientific) with an Accucore aQ C18 column (100 × 2.1 mm, 2.6 µm granulometry, Thermo Fischer Scientific) was used to separate MFN and TPs of all three investigated micropollutants. Quantification was performed by triple quadrupole mass spectrometry (MS/MS, TSQ Quantiva, Thermo Fisher Scientific) ^4,38^.

The contribution of biotic degradation $\left({\varvec{B}},{\varvec{t}}\boldsymbol{ }\boldsymbol{\%}\right)$ to overall micropollutant dissipation at the SWI at time *t* (Eq. 1) was determined as described previously^37^ from the relative dissipation *D,t* (100 – remaining proportion) in biotic (***D***_***biotic***_***, t*** %*)* and abiotic (***D***_***abiotic***_***, t*** %*)* microcosms.

1 $${\varvec{B}},{\varvec{t}}\boldsymbol{ }\boldsymbol{\%}={({\varvec{D}}}_{{\varvec{b}}{\varvec{i}}{\varvec{o}}{\varvec{t}}{\varvec{i}}{\varvec{c}}},{\varvec{t}}\boldsymbol{ }\boldsymbol{\%})-{({\varvec{D}}}_{{\varvec{a}}{\varvec{b}}{\varvec{i}}{\varvec{o}}{\varvec{t}}{\varvec{i}}{\varvec{c}}},{\varvec{t}}\boldsymbol{ }\boldsymbol{\%})$$

Dissipation rate *k* and half-lives (DT_50_) were determined from first-order kinetics (Eq. 2) with *C*_*t*_ the remaining concentration at time *t* and *C*_0_ the initial concentration (Table 1). Confidence intervals (CI 95%) were calculated with a z-score of 1.96 to compare* k* values across conditions and corrected by sample size $\sqrt{{\varvec{n}}}$ (Eq. 3).

2 $$C_{t} = C_{0} . e^{ - kt}$$

3 $$CI = \pm \frac{z - score*\sigma }{{\sqrt n }}$$

**Table 1:** Dissipation constants for MFN, MET, and TER in single (ONE) and multi-contamination (MIX) experiments under biotic and abiotic conditions.

Contaminant	Contamination type	Time (days)	D_abiotic,t, system_ (%) ± SD	D_biotic,t, system_ (%) ± SD	B_compound,t, system_ (%) ± SD	K_abiotic_ ± SE (day^−1^)	Estimated DT50_abiotic_ range (days)	K_biotic_ ± SE (day^−1^)	Estimated DT50_biotic_ range (days)	CI_95%_ K_abiotic_	CI_95%_ K_biotic_
MFN	ONE	0	0 ± 24	0 ± 38	0 ± 45	0.019 ± 0.004 (n = 10)	[30 : 46]	0.060 ± 0.011 (n = 13)	[10 : 14]	[0.017 : 0.021]	[0.054 : 0.066]
		15	33 ± 26	19 ± 53	-14 ± 59						
		30	40 ± 1	79 ± 24	39 ± 25						
		50	50 ± 29	95 ± 5	45 ± 29						
		70	77 ± 8	97 ± 2	20 ± 8						
	MIX	0	0 ± 0	0 ± 5	0 ± 5	0.013 ± 0.006 (n = 9)	[36 : 99]	0.059 ± 0.008 (n = 15)	[10 : 14]	[0.009 : 0.017]	[0.055 : 0.063]
		15	43 ± 42	47 ± 8	4 ± 43						
		30	60 ± 53	67 ± 15	6 ± 55						
		50	57 ± 4	93 ± 10	36 ± 10						
		70	71 ± 5	97 ± 4	27 ± 6						
MET	ONE	0	0 ± 2	0 ± 13	0 ± 13	0.060 ± 0.010 (n = 10)	[10 : 14]	0.045 ± 0.004 (n = 15)	[14 : 17]	[0.054 : 0.066]	[0.043 : 0.047]
		15	9 ± 9	14 ± 3	5 ± 9						
		30	68 ± 26	83 ± 5	15 ± 26						
		50	96 ± 1	91 ± 3	-5 ± 3						
		70	99 ± 0	94 ± 1	-5 ± 1						
	MIX	0	0 ± 7	0 ± 8	0 ± 11	0.072 ± 0.008 (n = 10)	[9 : 11]	0.054 ± 0.003 (n = 15)	[12 : 14]	[0.067 : 0.077]	[0.052 : 0.056]
		15	36 ± 4	67 ± 6	31 ± 7						
		30	46 ± 2	66 ± 7	20 ± 7						
		50	96 ± 0	93 ± 2	3 ± 2						
		70	99 ± 1	98 ± 0	1 ± 1						
TER	ONE	0	-	0 ± 10	-	0.006 ± 0.001 (n = 8)	[99 : 139]	0.005 ± 0.001 (n = 14)	[116 : 173]	[0.005 : 0.007]	[0.004 : 0.006]
		15	0 ± 9	8 ± 9	-1 ± 8						
		30	-2 ± 5	25 ± 8	27 ± 9						
		50	19 ± 8	28 ± 4	9 ± 9						
		70	23 ± 7	27 ± 3	4 ± 8						
	MIX	0	-	0 ± 4	-	0.009 ± 0.004 (n = 6)	[53 : 139]	0.005 ± 0.002 (n = 12)	[99 : 231]	[0.006 : 0.012]	[0.004 : 0.006]
		15	0 ± 1	31 ± 5	31 ± 5						
		30	-	39 ± 0	-						
		50	3 ± 19	43 ± 10	40 ± 21						
		70	40 ± 16	35 ± 7	-5 ± 17						

*B*: Biodegradation fraction; SD: error was estimated based on error propagation based on 1 σ; *k*: Dissipation rate estimated by the linear form of the first-order kinetic model ln Ct = ln C0^−kt^; SE: Standard error obtained from regression analysis of the first-order kinetic model; *DT50*: Estimated half-life of the compound.

CI_95%_ confidence interval of k calculated with a Z-score of 1.

### Prokaryotic composition analysis

#### DNA extraction

Environmental DNA was extracted from sediment and water at day 0 and day 70 for each condition using the DNeasy PowerSoil Pro Kit (QIAGEN) according to the manufacturer’s instructions. DNA concentrations were determined by fluorometry using Qubit dsDNA HS and BR kits (Thermofisher Scientific). DNA preparations were stored at − 20 °C. DNA concentrations ranged from 52 to 276 ng µL^−1^ (146 ± 76 ng µL^−1^) for sediment samples and from < 0.01 (u.d.l) to 188 ng µL^-1^ (67 ± 56 ng µL^−1^) for water samples (Table S4).

#### Amplicon sequencing and processing

The hypervariable V3−V4 region of the 16S rRNA gene was PCR amplified with the Pro341f/Pro806r primer pair targeting both bacteria and archaea^43^. Barcoded amplicon sequencing (paired-end 250 bases) was performed with the NovaSeq PE250 sequencing platform (Novogene, Cambridge, United-Kingdom). Library preparation for sequencing was outsourced to Novogene. Raw data were trimmed, filtered and denoised using the DADA2 pipeline^44^. Reads with an expected error number (E) of two or more were discarded. For each read, the error probabilities for each base, derived from quality scores, were summed. This sum enabled to calculate the expected E score, representing the average number of errors expected across identical reads. Potential chimeric reads were identified by comparing each read to others. A read was flagged as chimeric if it could be well-explained by combining two other, more abundant reads from the dataset. Obtained sequences were clustered at 100% identity, yielding a total of 26,354 Amplicon Sequence Variants (ASVs). Each ASV was annotated by applying QIIME2’s classify-sklearn algorithm on the Silva database (version 138, December 2019). Good’s coverage values indicated that sequencing depth exceeded 97.7% (average 99.2 ± 0.4%). ASVs present in only one of the triplicate samples of a given condition were discarded and data analysis was carried out based on the two other replicates, yielding 3,146 ASVs for taxonomic analysis. Taxonomic assignment decreased at finer taxonomic levels, with 1.1% of taxa remaining unassigned at the Phylum level, followed by 1.6%, 4.1%, 9.8%, 31.2%, and 92.8% at the class, order, family, genus, and species levels, respectively (Fig. S3). Unassigned ASVs were grouped by affiliation to the most precise taxonomic level available. Rarefaction curves for Chao1, Simpson, Pielou’s evenness, and Shannon indices (Figs. S4 and S5) indicated sufficient depth sequencing for all samples.

#### Data analysis

ASV sequences were analysed in R (version 4.3.1). Richness metrics (observed, Chao1, ACE), evenness indices (Camargo, Pielou, Simpson), and diversity measures (Shannon and Simpson) were computed (Table S5). Bray–Curtis matrices and dendrograms were generated employing the ‘phyloseq’ package. Analyses of similarities (NPMANOVA) were conducted at the ASV level with the ‘adonis2’ package.

Taxon-level mean fold change (FC) in relative abundance were calculated for each sample relative to its corresponding control (CTRL), i.e., the non-spiked sample for the same matrix and timepoint as the sample of interest at all taxonomic levels according to Eq. (4). In cases where a sample had one null abundance measurement among the three replicates, it was treated as a duplicate. A correction was applied, involving the addition of 0.001 to both the numerator (n) and denominator (d) (Eq. (4)), to prevent division by zero and avoid undefined or infinite results.

4 $$FC_{taxon, sample} = \left( {\frac{{mean(_{taxon, sample} ) + 0.001}}{{mean(_{taxon, control} ) + 0.001}}} \right) = \frac{n}{d} ;\quad n = d*FC_{taxon,sample}$$

A positive FC indicates an increase in relative abundance of a particular taxon when compared to the control condition. Log_10_ FC heatmaps were obtained after logarithmic transformation of FC values using “dplyr”, “tidyr”, “phyloseq”, “tibble”, and “heatmapply” packages.

The effects of individual micropollutants MFN, MET or TER (‘ONE experiments’) were compared with those of the micropollutant mixture (‘MIX experiment’) to investigate the occurrence of different types of interactions between micropollutants: additivity, antagonism, and synergism. Fold Change (FC) values were converted according to Eq. (5) into interaction coefficient (IC) values to assess the change in relative abundance of a taxon from the control to the sample of interest. The definition of ICs allowed for the evaluation of taxon-level mean fold changes (Eq. 4) while minimising potential biases in further calculations. For instance, if the abundance of a taxon decreases in both condition A and B compared to the control, resulting in FC < 1 (FC_A_ = 0.4, FC_B_ = 0.6), the sum of individual effects would yield a FC of 1, indicating no overall effect when summing the effects.

5 $$I{C}_{taxon,sample}=\left(\frac{n-d}{d}\right)=\left(\frac{d*F{C}_{taxon,sample}-d}{d} \right)=F{C}_{taxon,sample}-1$$

To evaluate the sum of micropollutant effects, the sum of IC values obtained for individual ‘ONE experiments’ with MFN, MET and TER was defined as *IC*_*taxon,ADD*_ (Eq. 6):

6 $$For \,sample_{k} ,i.e., MFN, MET, and TER : IC_{taxon,ADD} = \mathop \sum \limits_{k = 1}^{3} \left( {IC_{{taxon, sample_{k} }} } \right)$$

*IC*_*taxon,ADD*_ values were constrained to a minimum value of $-$ 1, which implies the absence of the considered taxon in all ‘ONE experiments’.

*IC*_*taxon,ADD*_ values were then compared for each taxon with IC values of the corresponding MIX experiment (*IC*_*MIX*_). A conservative uncertainty of ± 64% was associated with IC values basing on the third quartile (Q3) of ASV relative abundances from replicate experiments (Fig. S6). Thus, when *IC*_*MIX*_ ± 64% for a given taxon overlapped with *IC*_*ADD*_ ± 64%, the interaction of micropollutant effects was considered to be additive. However, when *IC*_*MIX*_ ± 64% exceeded *IC*_*ADD*_ ± 64%, interaction of micropollutants was considered synergistic. Conversely, when *IC*_*MIX*_ ± 64% was smaller than *IC*_*ADD*_ ± 64%, interaction of micropollutants was classified as antagonistic. Within the antagonistic category, two cases were identified without individual quantification: (i) “repressing” when *IC*_*MIX*_ < *IC*_*ADD*_, and (ii) “opposing” when *IC*_*MIX*_ and *IC*_*ADD*_ were of opposite sign, i.e., positive in *IC*_*ADD*_ and negative in *IC*_*MIX*_, or the reverse.

The contribution of individual micropollutants to the additive model at the Phylum and ASV levels was evaluated based on the proportion of taxa exhibiting the least difference between the observed FC scores of individual contaminants (MFN, MET, TER) and the mixture (MIX). In cases where two micropollutants ranked equally, they were defined as a 'binary combination' (i.e., MFN_MET, MFN_TER, and MET_TER).

Statistical tests for significance (*p* ≤ 0.05), including NPMANOVA, Wilcoxon, Dunn with Benjamini–Hochberg adjustment, Kruskal–Wallis, and the computation of confidence intervals, were conducted as required. For multiple comparisons, clusters were defined as replicate samples (n = 3) sharing a specific set of variables.

In the initial NPMANOVA to analyse the main effects of matrix, time, and contamination, a significant interaction term was identified as a potential confounding factor. To address this issue, permutations were restricted using the 'strata' argument for subsequent NPMANOVA analyses.

## Results and discussion

Laboratory microcosms mimicking the sediment–water interface of river surface waters allowed to investigate the dissipation of metformin (MFN), metolachlor (MET) and terbutryn (TER), spiked individually or as a mixture, and associated effects on prokaryotic communities. Unlike sediment–water interfaces in natural rivers, microcosms were not replenished with nutrients, so that transformation products may accumulate or undergo further degradation. Biological activity did not change significantly throughout the experiment as assessed by fluorescein diacetate transformation^42^ (Kruskal–Wallis, *p* = 0.17, Fig. S7).

Microcosms were also monitored in terms of chemical parameters throughout the experiment, and procaryotic composition was analysed at initial and final (day 70) timepoints. All microcosms displayed stable oxic conditions (8 ppm) and pH conditions (8 ± 1) (Table S6). Values of electric conductivity and concentrations of the major elements (NH_4_^+^; Na^+^; K^+^; Mg^2+^; Ca^2+^; Cl^-^; NO_3_^-^; SO_4_^2-^; PO_4_^2^) varied across conditions (Table S6). Since concentrations were derived from random sampling for each condition, statistical comparisons were not feasible. Biotic microcosms showed similar TOC levels as the original river water (5 ± 7 ppm), but with lower phosphate concentrations (0.01 ± 0.01 *vs* 2.94 mmol) and conductivities (Table S6). TOC was lower in biotic microcosms than in abiotic (autoclaved) microcosms (141 ± 123 ppm), suggesting DOC release to the water phase upon autoclaving as previously observed^45^.

### Dissipation and transformation of micropollutants at the sediment–water interface

**Figure 1:**
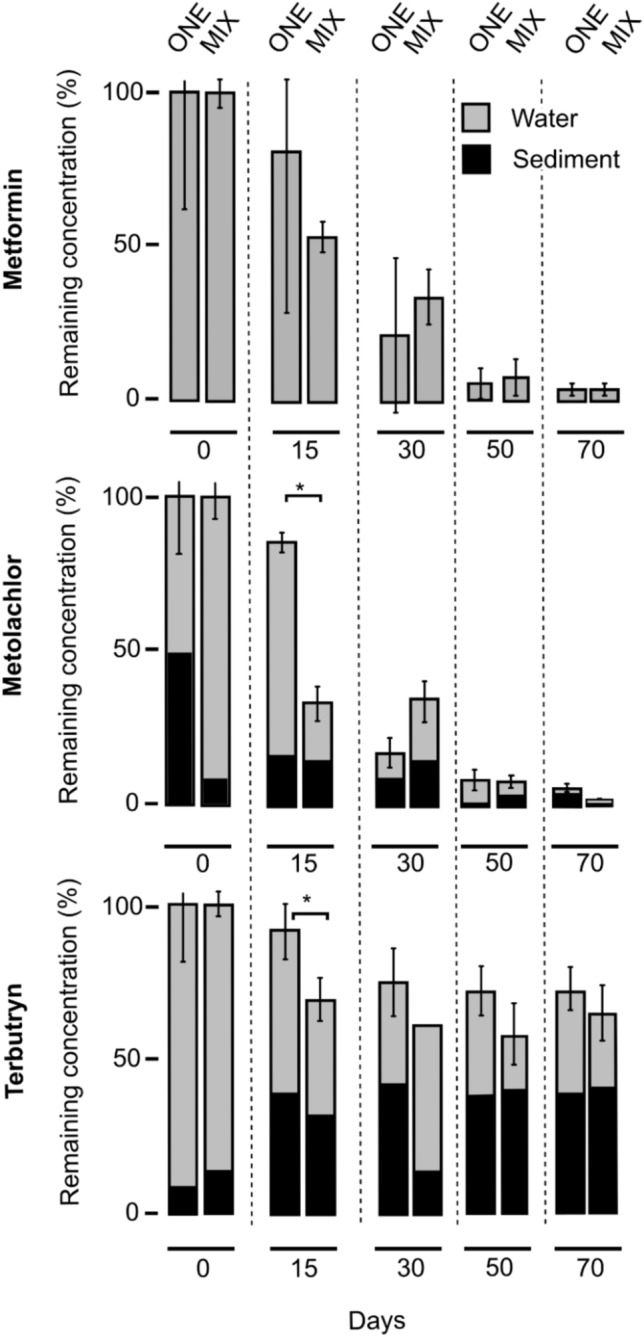
Distribution and dissipation of metformin, metolachlor and terbutryn in water and sediment phases in single (ONE) and multi-contamination (MIX) biotic experiments.

**Figure 2:**
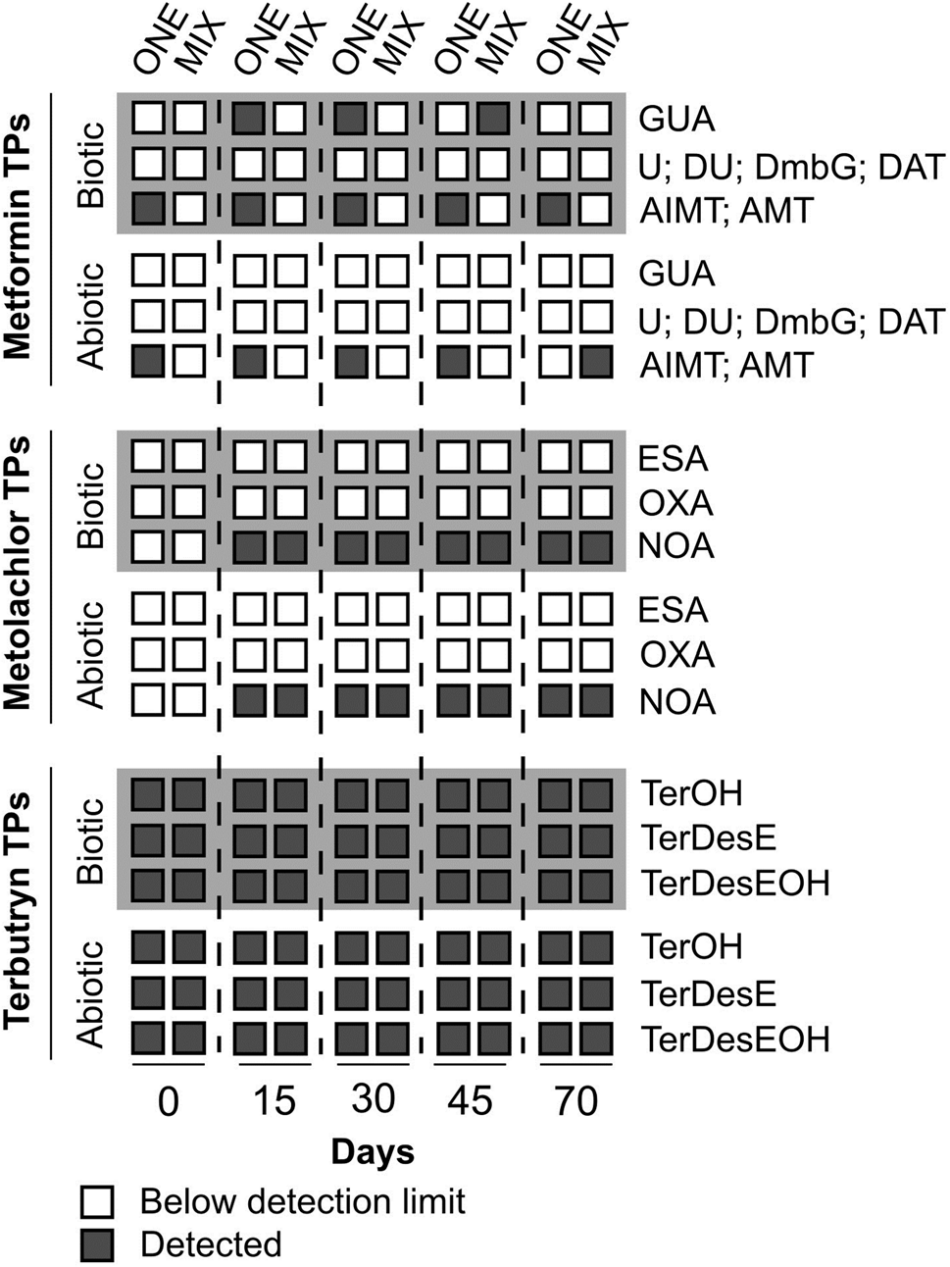
Transformation products (TPs) detected over time in biotic and abiotic experiments : guanylurea (GUA); urea (U); dimethylurea (DU); dimethylbiguanide (DMbG); 2,4-diamino-1,3,5-triazine (DAT); 4-amino-2-imino-1-methyl-1,2-dihydro-1,3,5-triazine (AIMT); 2-amino-4-methylamino-1,3,5-triazine (AMT); metolachlor ethanesulfonic acid (ESA); metolachlor oxanilic acid (OXA); metolachlor N-oxa-ethanesulfonicacid (NOA); 2-hydroxy-terbutryn (TerOH); desethyl-terbutryn (TerDesE); desethyl-2-hydroxy-terbutryn (TerDesOH).

MFN, MET and TER showed distinct partitioning in line with their physico-chemical properties between the sediment and the water phase (Fig. 1). The highly hydrophilic MFN was primarily found in water, and the more hydrophobic TER predominantly partitioned to the sediment, with MET showing intermediate behaviour (Fig. 1 and Table S7). This affected micropollutant dissipation dynamics, half-lives (DT_50_) (Table 1) and formation of transformation products (TPs) (Fig. 2 and Table S2). Micropollutant dissipation was similar when spiked individually (ONE experiments) or as part of a mixture (MIX experiment) for all three investigated micropollutants (Table S7).

#### Micropollutants distribution

MFN is a highly hydrophilic compound with a low octanol–water partitioning constant (logP < $-$ 2.48)^46^ and soil adsorption coefficient (K_oc_ < 20 L g^−1^)^47^. Unlike what was observed in a previous report^46^, metformin was only detected in the water phase of microcosms (Fig. 1 and Table S7). Absence of significant sorption to the sediment does not preclude passive water exchange between the water column and interstitial sediment water, allowing transformation of MFN in both water and sediment depending on the prevailing conditions and microbial activity in each compartment.

Unlike MFN, MET and TER partitioned between water and sediment (Table S7). MET partitioned equally, in line with its higher octanol–water and soil adsorption constants (LogP = 2.9; K_oc_ = 33 ± 9 L g^−1^)^23^. TER primarily partitioned to the sediment, consistent with its octanol–water partitioning and soil adsorption constants (LogP = 3.4; K_oc_ = 560 ± 240 L g^−1^)^48,49^.

#### Abiotic dissipation and biodegradation

*Metformin* MFN was unique among the three investigated micropollutants in showing significantly different dissipation between biotic and abiotic conditions (Table 1). MFN was the only chemical that underwent significant biodegradation, as its dissipation rates were significantly higher under biotic conditions (CI_95%_ k_abiotic_ = [0.009 to 0.021] day^-1^; CI_95%_ k_biotic_ = [0.054 to 0.066] day^−1^; Table 1). Evidence for microbial biodegradation of MFN has been abundantly documented, and bacteria growing with this compound as carbon and/or nitrogen source were reported recently^50–53^. Under biotic conditions, guanylurea, the major transformation product of MFN, appeared transiently at days 15 and 30 in ONE experiments, and at day 50 in the MIX experiment (Fig. 2). Guanylurea is utilised for growth by some bacteria with guanylurea hydrolase^54^. Other potential MFN transformation products dimethylguanidine, dimethylbiguanide, 2,4-diamino-1,3,5-triazine, dimethylurea or urea were not detected, suggesting that they were not produced or rapidly metabolised.

*Metolachlor* In the environment, MET undergoes photolysis^55^, hydrolysis^56^, and biodegradation^15^, with formation of potentially toxic transformation products (TPs). Also in contrast to MFN, dissipation rates of MET (Fig. 1) remained similar under biotic and abiotic conditions (Table 1). Patterns of detected TPs (Fig. 2) were similar under biotic and abiotic conditions suggesting that abiotic transformation processes were prominent (Table 1). Different pathways are known for MET degradation that involve abiotic and biotic transformations of the same TPs^57^. For example, formation of MET N-oxaethanesulfonic acid (NOA) from MET ethanesulfonic acid (ESA) was reported in soil^58^. Here, only NOA was detected, while ESA or the other major oxanilic acid derivative of metolachlor (OXA) were not identified (Fig. 2).

*Terbutryn* TER was the most recalcitrant micropollutant in our experiments (Fig. 1 and Fig. 2). TER contamination persisted, with over 60% TER remaining after 70 days (Fig. 1, Table S7). Estimated half-lives for TER (53–231 days, Table 1) are in agreement with previously reported values for aerobic river sediment^28^ and groundwater (193–644 days)^59^. Transformation products^60^ 2-hydroxy-terbutryn (TerOH), desethyl-tebutryn (TerDesE), and desethyl-2-hydroxyterbutryn (TerDesEOH) were detected in all TER microcosms (Fig. 2 and Table S2).

#### Effect of multicontamination on micropollutant transformation processes

Overall, the presence of other micropollutants did not affect the dissipation processes of MFN, MET, and TER. MFN dissipation remained unaffected by the two other micropollutants (Fig. 1; Table 1). The delayed and transient detection of guanylurea in the MIX experiment compared to microcosms spiked exclusively with MFN is of particular interest. Organisms involved in the degradation of MFN and guanylurea may have been adversely affected by co-occurrence of MET and/or TER, resulting in transient guanylurea accumulation in MIX experiment. In addition to guanylurea, minor transformation products 2-amino-4-methylamino-1,3,5-triazine (AMT) and 4-amino-2-imino-1methyl-1,2-dihydro-1,3,5-triazine (AIMT)^30^ were identified in ONE experiment but remained undetected in the MIX experiment (Fig. 2). This difference could be due to the high limits of quantification for AMT and AIMT. Half-lives for MET displayed a slight yet statistically significant increase in ONE experiments containing MET alone (14–17 days) as compared to the MIX experiment (12–14 days) (Table 1). This contrasts with previous reports of slower dissipation of micropollutants such as metolachlor, mesotrione, atrazine, bentazone, glyphosate and diflufenican in the presence of other contaminants^15,58,61^. Here, dissipation rates and transformation patterns of TER were unaffected by the presence of the other two micropollutants (Figs. 1, 2, Table 1). The transformation products TerOH, TerDesE, and TerDesEOH were consistently detected in ONE and MIX experiments, indicating similar dissipation processes under these two conditions.

A previous study^30^ used sediment–water microcosms spiked with a high number (56–80) of micropollutants reported minimal changes in individual biodegradation rates and suggested that changes might become more significant as micropollutant concentrations increase^30^. In our study, only MFN biodegradation was significant. Since we used an initially high concentration of MFN, MFN biodegradation may not be significantly affected by the presence of metolachlor (MET) and terbutryn (TER), regardless of their concentration in the environment.

### Factors affecting prokaryotic communities in sediment–water microcosms

Prokaryotic diversity in microcosms was assessed by sequencing PCR amplicons of the 16S ribosomal gene V3–V4 variable region at initial and final time points. Overall, the sediment showed significantly higher prokaryotic richness, evenness, and diversity indices than the water phase (Fig. S8 and Table S5). Previous studies also documented distinct diversity from sediment and water issued from the same aquatic ecosystem^48^. Diversity metrics in sediment and water phases both significantly decreased over time (Fig. S8), but significant effects of micropollutants were not observed (Table S5, Dunn with Benjamini–Hochberg adjustment, *p* > 0.1).

**Figure 3:**
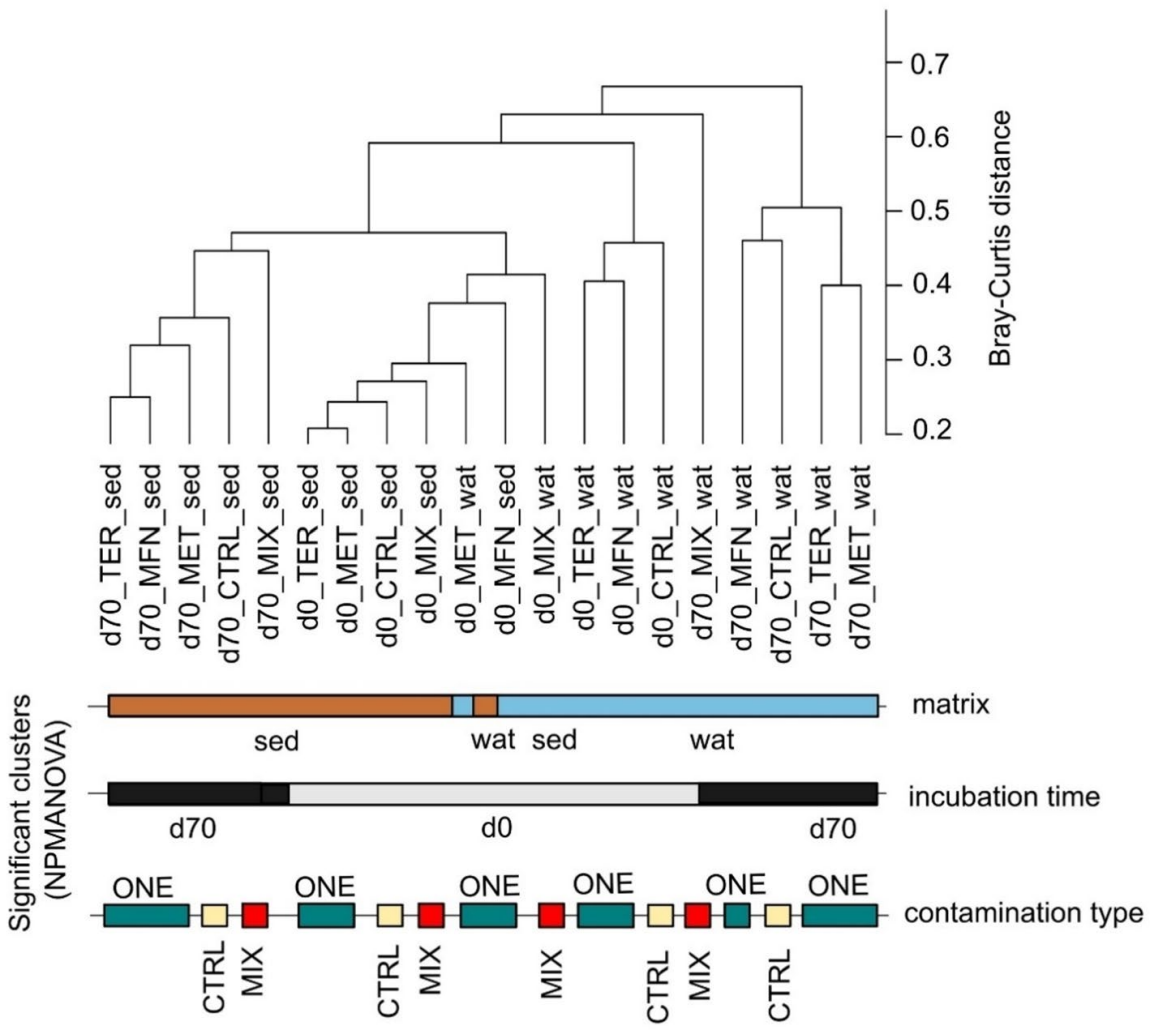
Dendrogram (mean Bray–Curtis distances) with clustering according to matrix (sediment (sed) or water (wat)), timepoint (d0/d70), and contamination type (ONE/MIX/CTRL).

Some studies reported significant changes in bacterial communities exposed to micropollutants^32,33^, but this has not always been observed ^34,35^. Site-specific factors and characteristics may modulate the effect of micropollutant exposure on alpha diversity. The complexity of natural environments, with varying exposure scenarios and interacting factors, may also complicate the identification of the primary determinants of changes in prokaryotic community composition^62^. Nevertheless, micropollutants may significantly affect the composition of prokaryotic communities through their intrinsic toxicity^63–66^. Here, we were able to analyse differences in amplicon sequence variant (ASV) data in laboratory microcosm samples and to a certain extent, tease apart the contribution of different factors to changes in prokaryotic diversity. Using distance matrices (Fig. 3) and statistical analysis (Tables S8 and S9), the factors affecting bacterial (Table S8) and archaeal (Table S9) prokaryotic community composition were thus ranked in order of importance, with matrix ranking first, followed by time and then contamination type.

**Figure 4:**
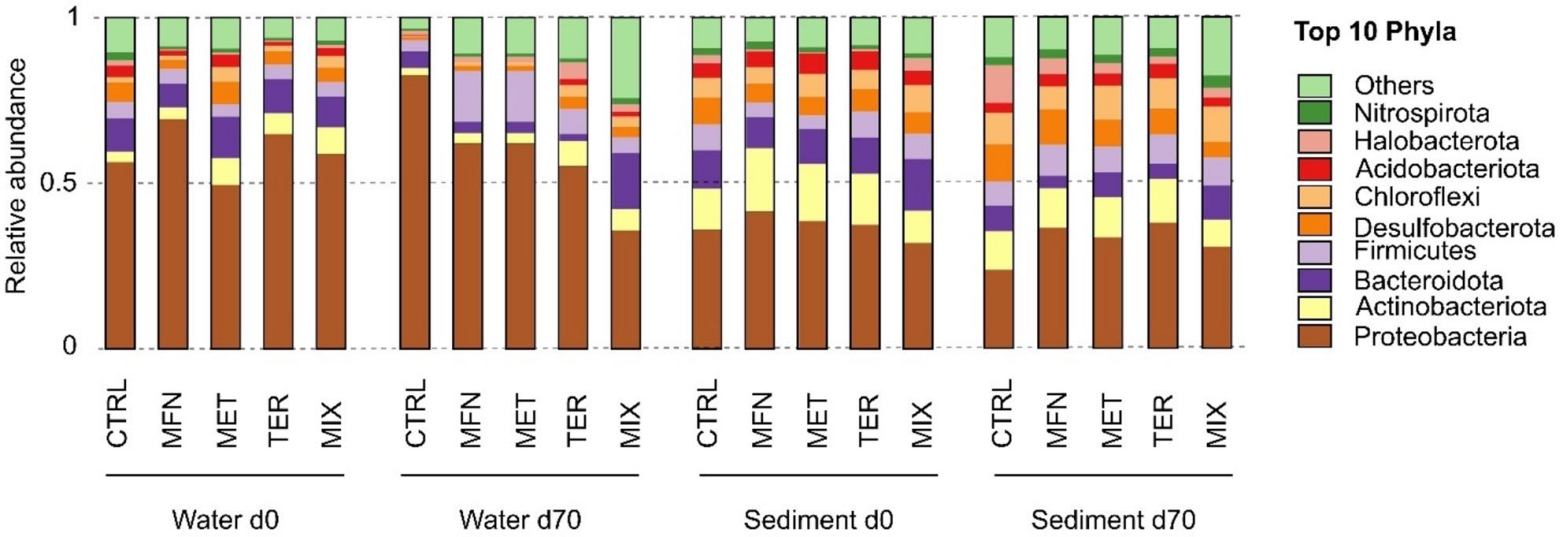
Distribution of the top 10 phyla across CTRL, MFN, MET, TER, MIX conditions from sediment and water phase at d0 and d70.

Overall, phase, timepoint and contamination type as well as interactions between these three factors (Table S8) accounted for over 67% of the observed variability in bacterial community composition, leaving a residual R^2^ of 33%. This residual variation represents unidentified experimental variability during set-up and among replicates despite rigorous initial sediment homogenization, and as expected in the OECD 309 test^30^. The observed clustering (Fig. 3) reveals substantial disparities in prokaryotic composition between sediment and water phases (NPMANOVA: R^2^ = 0.13; F = 15.71; *p* = 0.0001). Similar patterns were observed for archaeal communities (Table S9). Differences between sediment and water were already documented previously^67^, and likely find their origin in qualitative and quantitative differences in nutrient availability and organic substrates in the two environments. For instance, the abundance of Proteobacteria significantly differed between the sediment phase (34.40 ± 5.46%) and the water (59.92 ± 17.18%) (Table S10 and Fig. 4).

Incubation time was another important factor affecting bacterial community composition (NPMANOVA: R^2^ = 0.13; F = 15.34; *p* = 0.0001, Tables S8 and S11). Again, similar patterns were observed for archaeal communities (Table S9). As expected and also observed previously^68^, prokaryotic community composition adjusted to laboratory conditions and also led to the development of initially undetected taxa during incubation, e.g., here for Iainarchaeota in the water phase and WS4 in the sediment. A trend towards increase in the proportion of Archaea across all microcosms was also observed, i.e., from 3.4 ± 3.4% to 6.7 ± 5.9% in the sediment and from 1.3 ± 1.1% to 6.2 ± 5.0% in the water phase over the 70-day incubation period (Table S11).

The composition of prokaryotic communities was also affected by the type of contamination as evidenced by significant difference observed across CTRL, MFN, MET, TER, and MIX experiments (NPMANOVA: R^2^ = 0.12; F = 3.51; *p* = 0.0001, Table S8). We examined the effect of contamination in conjunction with time (day 0 or day 70) (Table S12). To prevent confounding effect of the phase, separate analyses were conducted for sediment and water samples at day 0 and day 70. No significant differences were observed across CTRL, MFN, MET, TER, and MIX experiments (Table S12).

We also evaluated whether observed changes in prokaryotic communities in the MIX experiment differed from the cumulative changes observed in MFN, MET, and TER experiments in sediment, water or the entire dataset at day 0 and day 70 (Table S13). The MIX experiment showed a significantly distinct bacterial community composition at the ASV level compared to the combined ONE experiments. However, there was no significant difference (*p* = 0.10) between MIX and CTRL experiments, likely because of the limited sample size of each CTRL and MIX subgroup (n = 3, Table S13). Analyses of archaeal community composition did not highlight any significant differences between experimental conditions (Table S14).

**Figure 5:**
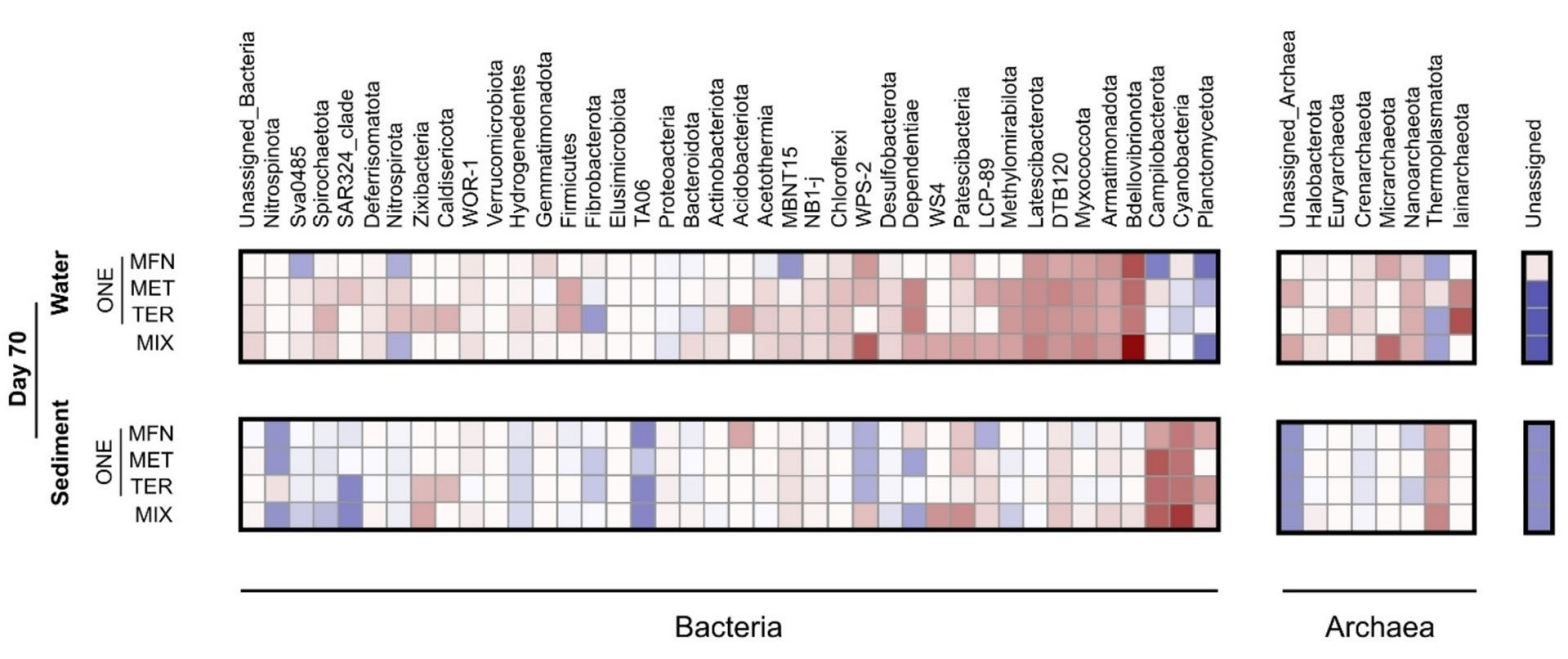
Heatmap of log_10_FC in relative abundance of phyla in ONE and MIX experiment compared to CTRL experiments for sediment and water phases at the end of microcosm incubations (day 70). Phyla showing an increase in relative abundance (positive log_10_FC) compared to CTRL experiments are shown in red, and phyla showing a decrease (negative log_10_FC) in blue.

Finally, in an attempt to identify specific changes in prokaryotic communities associated with micropollutant exposure, we compared changes in relative abundances in MFN, MET, TER, and MIX experiments with those of control experiments without contamination (CTRL). A large proportion of taxa varied in relative abundance across all taxonomic levels from ASVs to phylum (Figs. 4, and 5). It is well-documented that contamination can have either positive or negative affects on the relative abundance of different taxa^69^. Accounting for all taxonomic levels, 63 ± 2% of phyla showed an increase in relative abundance (FC > 1), and 37 ± 2% a decrease in relative abundance (FC < 1) at the end of the experiment compared to the corresponding control (CTRL) (Table S15).

Some taxa showed clear responses to different micropollutants for a given phase. For example, in the water phase, the response of certain phyla such as Nanoarchaeota, Latescibacteria, and Bdellovibrionata was similar in ONE and MIX experiments (Fig. 5). Bdellovibrionota showed a marked increase in relative abundance in the MIX experiment compared to that observed in ONE experiments (average FC in ONE experiments = 262 ± 183, FC in MIX = 6253; Fig. 4). This enrichment of Bdellovibrionota (average FC in ONE experiments = 207 ± 174, FC in MIX = 304; Fig. 4), a phylum known for bacterial predation, suggests that part of the observed decrease in certain taxa may result from predation by Bdellovibrionota. In addition, the enrichment of Campilobacterota, a phylum containing human pathogens, suggests potential concerns regarding health risks associated with exposure to micropollutants.

Previous research already demonstrated that mixtures of micropollutants may significantly amplify the effects of individual micropollutants^15^. This suggests that the effects of micropollutant mixtures on prokaryotic communities may be difficult to extrapolate from the effects of individual micropollutants. This encouraged us to explore the occurrence of three possible types of micropollutant interactions: additivity, when the observed effect on a given taxon in the MIX experiment corresponds to the total individual effects in ONE experiments; antagonism, when it is less than the total of individual micropollutant effects; and synergism, when it exceeds the sum of individual micropollutant effects.

### Evidence for non-additive effects of micropollutants on prokaryotic communities

Non-additive effects of micropollutants on procaryotic communities may arise for several reasons. For example, increased proliferation of pollutant-tolerant strains producing key nutrients or factors may promote growth of specific taxa^70^. Conversely, production of antibiotics or toxic compounds by pollutant-tolerant taxa may have inhibitory effects on other taxa^71,72^.

**Figure 6:**
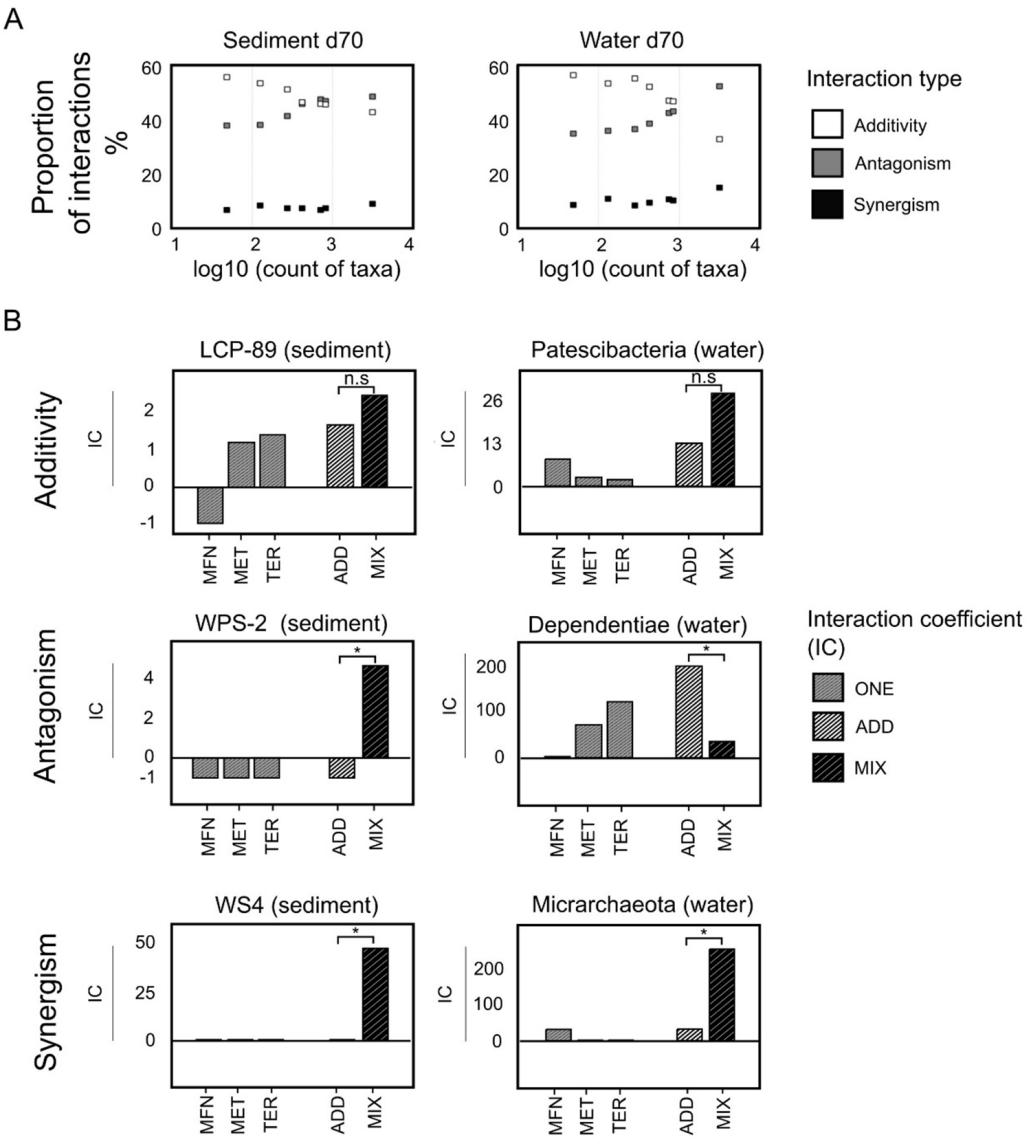
Interactions (additivity, antagonism, synergism) among micropollutant effects across taxonomic levels for sediment and water phases. (**A**) Proportion (percentage) of interactions as a function of number of taxa at different taxonomic levels (x-axis, from left to right: Phylum, Class, Order, Family, Genus, Species, ASV). To count taxa, we enumerated the taxa present across various taxonomic levels, and applied a log10 transformation to reduce graphical distance. (**B**) Examples of different interaction types at the Phylum level in sediment (left) and water (right). The relative error (RE) threshold for significance of differences in interaction coefficient (IC) values was set at ± 64%. (*) denotes statistically significant differences between IC scores predicted for the ADD model and observed in the MIX experiment (n.s., not significant).

Worthy of note identified interactions across various matrices were largely consistent at different taxonomic levels (Fig. 6A). As expected, additivity emerged as prevalent (33–57% of cases) in line with the conservative criterium chosen our study, i.e., differences within 64% relative error were considered non-significant. Antagonistic interactions involving either repressing or opposing effects (see Materials and Methods) also accounted for a substantial proportion (34–52%) of the total identified interactions. Synergistic interactions in the effects of micropollutants were noted for only a minor proportion of taxa, in the range of 5% to 15% of the total across all taxonomic levels from ASV to Phylum. The proportion of additive outcomes decreased at more precise taxonomic levels (Fig. 6A), with the Phylum level demonstrating the highest degree of additivity (57%), and the ASV level exhibiting the lowest (33%). This trend presumably originates in the larger number of taxa detected in the MIX experiment at the ASV level (Fig. 6A and Table S5).

To illustrate the proposed approach, Phylum LCP-89 is an example of a Phylum-level taxon responding to micropollutants in an additive way in the sediment (Fig. 6B). The sum of individual IC values of MFN, MET and TER (IC_ADD_ = 1.5 ± 1) did not differ significantly from that observed in the corresponding MIX experiment (IC_MIX_ 2.4 ± 1.5). It is worth noting that an IC_ADD_ value can be computed from the sum of positive and negative individual IC values as for Phylum LCP-89, or from the sum of IC values sharing the same sign, as exemplified by Patescibacteria in the water phase (Fig. 6B).

Potential antagonistic interactions for the effects of the micropollutant mixture were also identified. In such cases, IC values for the micropollutant mixture differed from the IC_ADD_ value computed from the added effects of the three individual micropollutants in two different ways, termed ‘antagonism (opposing)’ and ‘antagonism (repressing)’ (Fig. 6B, middle row). The WPS-2 phylum in the sediment phase is an example of 'antagonism (opposing),' with a negative calculated IC_ADD_ value (− 1.0 ± 0.6) and a positive observed IC_MIX_ (4.6 ± 2.9) (Fig. 6B). In contrast, Dependentiae in the water phase showed a lower IC_MIX_ value (35 ± 22) than the computed IC_ADD_ (193 ± 124), categorized as 'antagonism (repressing)'.

Examples of synergistic effects between pollutants, i.e., when IC values for the MIX experiment exceeded the summation of computed IC values in ONE experiments, include the case of Phyla WS4 in the sediment (IC_ADD_ = 0, IC_MIC_ = 47 ± 30) and Micrarchaeota in the water phase (IC_ADD_ = 30 ± 19, IC_MIX_ = 255 ± 163) (Fig. 6B).

**Figure 7:**
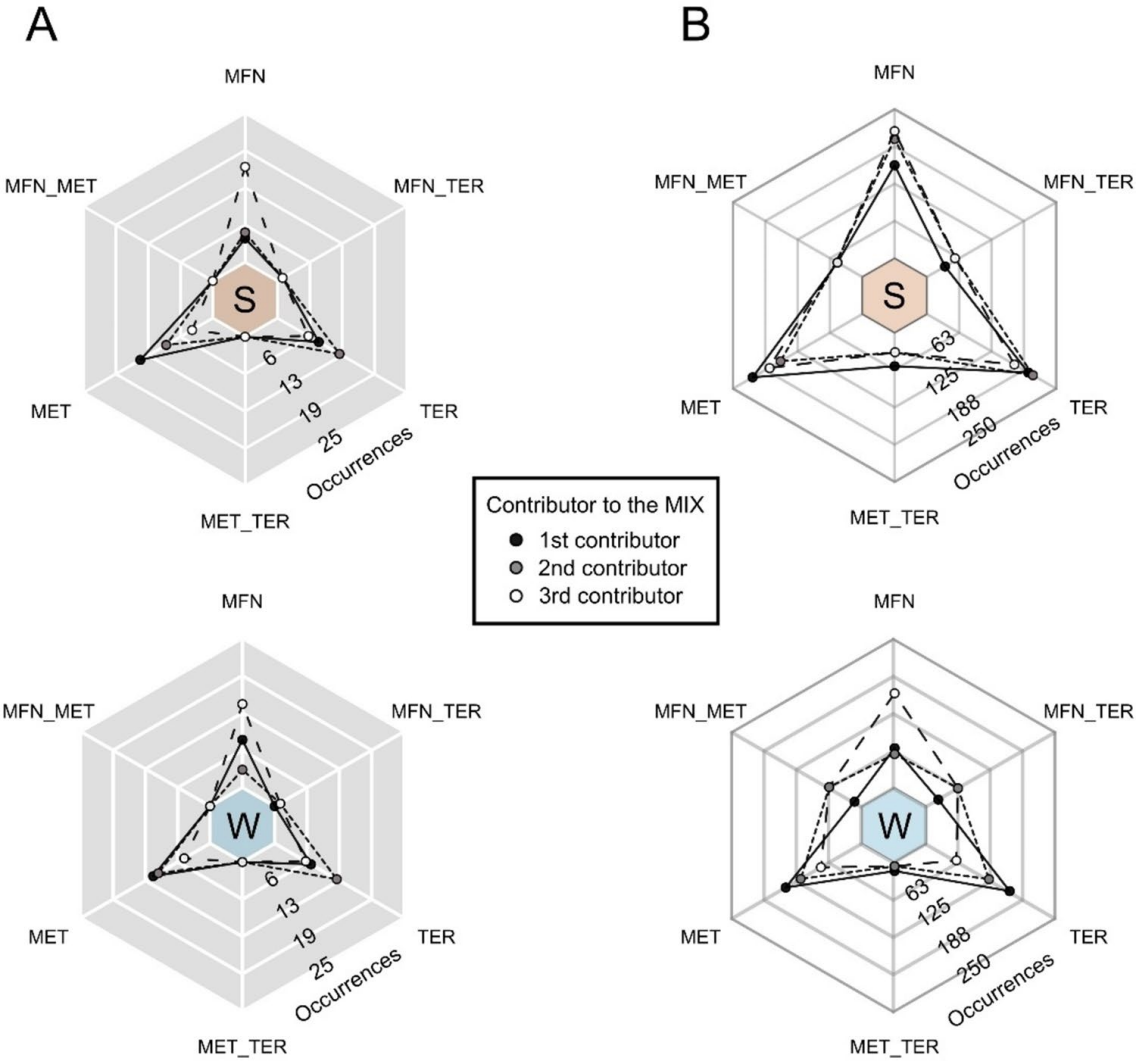
Contribution of micropollutants to the additive model at the Phylum (part **A**, grey) and ASV (part **B**, white) levels in sediment (S) and water (W) compartments. Micropollutants contributed individually (MFN, MET, TER) or in binary combination to equal degrees (MFN_MET, MFN_TER, and MET_TER, see Materials and Methods) to the observed effects in the MIX experiment.

Given the notable prevalence of additivity interactions, we conducted a ranking of individual micropollutant contributions to additivity. Micropollutants were assessed based on their FC scores in ONE experiments, comparing them to the FC scores of the MIX experiment (Fig. 7). MET and TER consistently emerged as the most frequent first and second contributors across the entire range of taxonomic levels (Fig. 7). MFN typically represented the smallest contributor, although differences were less pronounced in the sediment at the ASV level. MET and TER primarily target physiological processes not directly relevant to prokaryotic communities, although indirect effects of such pesticides may be envisaged^27,73^. In contrast, MFN and other pharmaceuticals are currently considered to primarily impact eukaryotes, although their transformation products may have indirect effects on procaryotes as well^21^.

Also worthy of note in this context, the minor effect of MFN as the least recalcitrant of the three micropollutants investigated in this study may result from its decrease in concentration over time compared to MTF and TER. This phenomenon warrants further investigation in future studies.

## Conclusions

Understanding the complex interplay of micropollutant dissipation, interactions among multiple micropollutants, and dynamics of prokaryotic community at the sediment–water interface poses a multifaceted challenge that requires consideration of micropollutant matrix-dependent bioavailability, toxicity, and transformation. In our study, laboratory microcosms were used to simulate the sediment–water interface under controlled conditions, and were spiked with three prominent micropollutants, either individually or as a mixture. Kinetic analysis of degradation and the formation of transformation products did not reveal significant effects of multicontamination on the dissipation of individual micropollutants. Similarly, the type of contamination did not significantly affect overall richness, evenness, or diversity of prokaryotic communities (Table S5). However, micropollutants notably affected the bacterial composition more than the archaeal composition. Specific taxa showed varying degrees of susceptibility to micropollutants depending on the matrix and type of contamination, evident across different taxonomic levels. Furthermore, significant deviations from the sum of individual micropollutant effects were detected when micropollutants were provided at the same concentration in a mixture.

We expect that the analytical framework established in this study may prove valuable for testing and prioritizing the biological effects of a specific compound within complex micropollutant mixtures. This approach has the potential to enhance the accuracy of risk assessments related to multi-contamination in aquatic ecosystems.

### Supplementary Information

Supplementary Information.

## Data Availability

Sequence data were deposited in the European Nucleotide Archive (ENA) at EMBL-EBI as BioProject PRJEB76198. The datasets generated and analysed in the study are available from the corresponding author upon reasonable request. Micropollutant concentration data can be found at https://github.com/ITESbiogeochem/Data_SYN.git.
