# Supplementary material for: Combined effects of micropollutants and their degradation on prokaryotic communities at the sediment–water interface
Source: Sci Rep. 2024 Jul 22;14:16840. doi: 10.1038/s41598-024-67308-y (PMC11263610; doi:10.1038/s41598-024-67308-y)
Supplement: Supplementary file 1 — Supplementary Information. [file 41598_2024_67308_MOESM1_ESM.docx]

***Supplementary Information***

**Combined effects of micropollutants and their degradation**

**on prokaryotic communities at the sediment-water interface**

Adrien Borreca^a,b^, Stéphane Vuilleumier^b^, Gwenaël Imfeld^a,^*

^a^ Institut Terre et Environnement de Strasbourg, UMR 7063 CNRS, ENGEES, Université de Strasbourg, 67000 Strasbourg, France.

^b^ Génétique Moléculaire, Génomique, Microbiologie, UMR 7156 CNRS, Université de Strasbourg, Strasbourg, France.

*Corresponding author: Gwenaël Imfeld (e-mail: [imfeld@unistra.fr](mailto:imfeld@unistra.fr))

Manuscript for Scientific Reports

Supplementary tables

Table S1. Studied micropollutants and their putative transformation products.

| **Name** | **Abbreviation** | **Nomenclature** | **Potential precursor** | **Purity** | **Provider** | **Comment** |
| --- | --- | --- | --- | --- | --- | --- |
| **Metformin** | MFN | C_4_H_11_N_5_ | - | ≥ 97% | Acros organics | - |
| **Metolachlor** | MET | C_15_H_22_ClNO_2_ | - | ≥ 98% | Sigma-Aldrich | - |
| **Terbutryn** | TER | C_10_H_19_N_5_S | - | ≥ 98% | Sigma-Aldrich | - |
| **Guanylurea** | GUA | C_2_H_6_N_4_O | MFN | ≥ 99% | Sigma-Aldrich | - |
| **Dimethylguanidine** | DMG | C_3_H_9_N_3_ |  | ≥ 99.5% | Acros organics | - |
| **Dimethylbiguanide** | DMbG | C_4_H_12_ClN_5_ |  | ≥ 98% | Acros organics | - |
| **Dimethylurea** | DU | C_3_H_8_N_2_O |  | ≥ 98% | Acros organics | - |
| **Urea** | U | CH_4_N_2_O |  | ≥ 99% | Acros organics | - |
| **2,4-Diamino-1,3,5-triazine** | DAT | C_3_H_5_N_5_ |  | ≥ 99% | Sigma-Aldrich | No available standard, use of melamine (MAM) as proxy ^26^ |
| **2-Amino-4-methylamino-1,3,5-triazine** | AMT | C_4_H_7_N_5_ |  |  |  |  |
| **4-Amino-2-imino-1-methyl-1,2-dihydro-1,3,5-triazine** | AIMT | C_4_H_6_N_4_O |  |  |  |  |
| **Metolachlor ethanesulfonic acid** | ESA | C_15_H_23_NO_5_S | MET | ≥ 95.0 % | Sigma-Aldrich | - |
| **Metolachlor oxanilic acid** | OXA | C_15_H_21_NO_4_ |  | ≥ 98.0 % | Sigma-Aldrich | - |
| **Metolachlor N-oxa-ethanosulfonic acid** | NOA | C_14_H_17_NNa_2_O_6_ |  | ≥ 98.0 % | TechLab | - |
| **Terbutryn-2-hydroxy** | TerOH | C_9_H_17_N_5_O | TER | ≥ 98% | HPC Standards GmbH | - |
| **Desethyl-terbutryn** | TerDesE | C_8_H_15_N_5_S |  | ≥ 98% | HPC Standards GmbH | - |
| **Desethyl-2-hydroxy-terbutryn** | TerDesEOH | C_7_H_13_N_5_O |  | ≥ 98% | HPC Standards GmbH | - |
| **Metformin-d6** | MFNd6 | C_4_H_5_d_6_N_5_ | - | ≥ 95% | Sigma-Aldrich | LC/MS-MS internal standard |
| **Metolachlor-d11** | METd11 | C_15_H_11_d_11_ClNO_2_ | - | ≥ 97% | Sigma-Aldrich | GC/MS internal standard |

Table S2. Analytical parameters with limits of detection (LoD) and limits of quantification (LoQ) for LC/MS-MS and GC/MS measurements. These parameters were evaluated using calibration curves, compounds were spiked directly in solvent.

|  |  |  |  |  |  |  |  |
| --- | --- | --- | --- | --- | --- | --- | --- |
|  | **Compound** | **Instrument** | **LoD** | **LoQ** | **Relative measurement error (%) at 95% confidence interval** | **Precursor** (polarity mode) | **Fragments** |
|  |  |  | **(µg L^-1^)** | **(µg L^-1^)** |  |  |  |
| **Parent molecule** | MFN | LC/MS-MS | 0.064 | 0.195 | 2.4 | 130 (+) | 126/60 |
|  | MET | GC/MS | 5.772 | 17.490 | 10.1 | - | 162/238 |
|  | TER | GC/MS | 15.793 | 47.859 | 11.2 | - | 185/226 |
|  |  |  |  |  |  |  |  |
| **MFN_TPs** | GUA | LC/MS-MS | 0.031 | 0.925 | 4.2 | 103 (+) | 60/86 |
|  | U | LC/MS-MS | 0.194 | 0.587 | 5.9 | 61 (+) | 44/- |
|  | DU | LC/MS-MS | 2.955 | 8.954 | 11.9 | 89 (+) | 72/46 |
|  | MAM | LC/MS-MS | 33.574 | 101.7385 | 31.4 | 127 (+) | 85/68 |
|  | DAT | LC/MS-MS | 16.922 | 512.794 | 55.1 | 112 (+) | 70/68 |
|  | DMbG | LC/MS-MS | 6.209 | 18.816 | 8.8 | 88 (+) | 71/46 |
|  |  |  |  |  |  |  |  |
| **MET_TPs** | ESA | LC/MS-MS | 2.121 | 6.427 | 3.5 | 328 (-) | 121/134 |
|  | OXA | LC/MS-MS | 2.639 | 7.998 | 2.9 | 278 (-) | 174/205 |
|  | NOA | LC/MS-MS | 1.738 | 5.268 | 3.7 | 328 (-) | 256/284 |
|  |  |  |  |  |  |  |  |
| **TER_TPs** | TerOH | LC/MS-MS | 7.315 | 22.168 | 11.7 | 212 (+) | 86/156 |
|  | TerDesE | LC/MS-MS | 2.728 | 8.266 | 10.1 | 214 (+) | 158/210 |
|  | TerDesEOH | LC/MS-MS | 2.800 | 8.485 | 6.1 | 184 (+) | 184/86 |

Table S3. Recovery rates for metformin, metolachlor, and terbutryn for QueChERS-MUSE and SPE extractions methods from river sediment. Uncertainty denotes standard deviation from triplicate experiments

| **Extraction methods** | **Metformin** | **Metolachlor** | **Terbutryn** |
| --- | --- | --- | --- |
| QueChERS-MUSE | non-significant (<10%) | 74 ± 4% | 67 ± 7% |
| SPE | - | 115 ± 23 % | 86 ± 17% |

Table S4. DNA quantification using Qubit dsDNA kit.

|  |  |  | **DNA concentration (ng µL^-1^)** | | |
| --- | --- | --- | --- | --- | --- |
| **Compound** | **Time** | **Replicate** | **Sediment** | **Water** |  |
| MET | t0 | a | 115 | 188 |  |
| MET | t0 | b | 218 | 128 |  |
| MET | t0 | c | 119 | 162 |  |
| TER | t0 | a | 276 | 9.42 |  |
| TER | t0 | b | 154 | 21.2 |  |
| TER | t0 | c | 147 | 12.2 |  |
| MFN | t0 | a | 109 | 20.6 |  |
| MFN | t0 | b | 63.4 | 1.2 |  |
| MFN | t0 | c | 161 | 6.4 |  |
| MIX | t0 | a | 190 | 25.08 |  |
| MIX | t0 | b | 192 | 177 |  |
| MIX | t0 | c | 137 | 26.2 |  |
| CTRL | t0 | a | 52.2 | 10.4 |  |
| CTRL | t0 | b | 248 | 0.8 |  |
| CTRL | t0 | c | 46.4 | 44.2 |  |
| MET | t70 | a | 129 | 6.1 |  |
| MET | t70 | b | 89 | 50.8 |  |
| MET | t70 | c | 91.2 | 5.2 |  |
| TER | t70 | a | 81.4 | 11.0 |  |
| TER | t70 | b | 116 | 0.01 |  |
| TER | t70 | c | 53.8 | 3.16 |  |
| MFN | t70 | a | 145 | 27 |  |
| MFN | t70 | b | 68.2 | 5.8 |  |
| MFN | t70 | c | 124 | 2.9 |  |
| MIX | t70 | a | 182 | 12.7 |  |
| MIX | t70 | b | 144 | 4.2 |  |
| MIX | t70 | c | 122 | 13.4 |  |
| CTRL | t70 | a | 72.6 | 14 |  |
| CTRL | t70 | b | 114 | 13.2 |  |
| CTRL | t70 | c | 150 | 21.2 |  |

Table S5. Sample size (i.e., number of samples in subgroups), richness, evenness, diversity indices and count of taxa at different taxonomic level across sediment and water samples, and subsequent subgroups of contaminants (CTRL, MFN, MET, TER, MIX) in sediment and water phases at day 70.

**
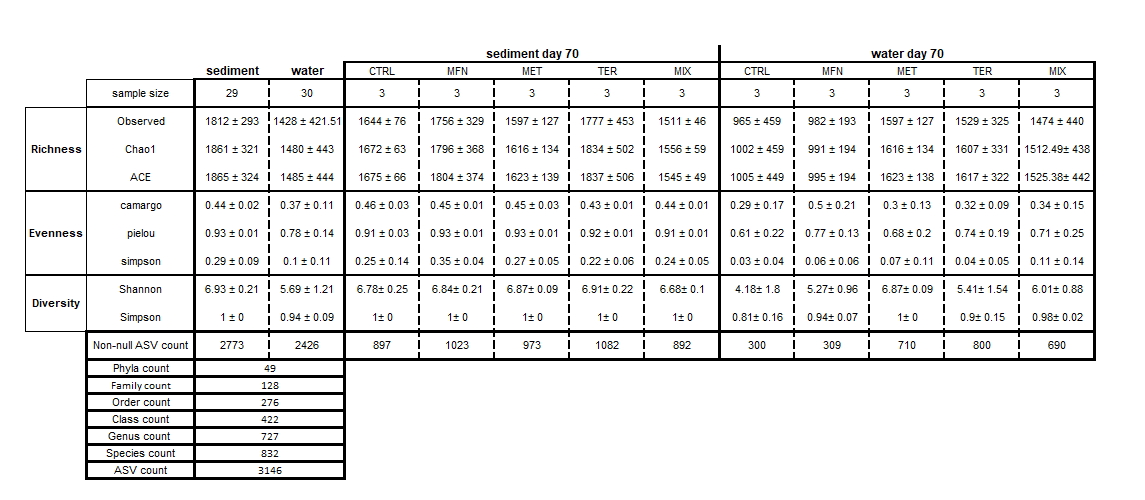
**

Table S6. Hydrochemistry of river water and the water phase at days 0 and 70 in biotic and abiotic microcosm experiments.

|  | **d0** | | **d70** | |  |
| --- | --- | --- | --- | --- | --- |
|  | **Abiotic** | **Biotic** | **Abiotic** | **Biotic** | **River** |
| NH_4_^+^ (mmol L^-1^) | 0.21 | 0.02 | 3.54 | 0.11 | 0 |
| Na^+^ (mmol L^-1^) | 0.31 | 0.26 | 0.34 | 0.23 | 0.49 |
| K^+^ (mmol L^-1^) | 0.18 | 0.03 | 0.25 | 0.14 | 0.14 |
| Mg^2+^ (mmol L^-1^) | 0.76 | 0.22 | 1.08 | 1.14 | 1.71 |
| Ca^2+^ (mmol L^-1^) | 2.29 | 0.19 | 1.89 | 1.76 | 3.95 |
| Cl^-^ (mmol L^-1^) | 0.18 | 1.38 | 0.29 | 0.21 | 1.33 |
| NO_3_^-^ (mmol L^-1^) | 0.01 | 0.21 | 0.02 | 0.04 | 0.80 |
| SO_4_^2-^ (mmol L^-1^) | 1.14 | 1.48 | 0.27 | 0.81 | 1.98 |
| PO_4_^3-^ (mmol L^-1^) | 0 | 0.22 | 0.01 | 0 | 2.89 |
| TOC (ppm) | 228 | 0.27 | 54.4 | 10.2 | 2.9 |
| Conductivity (S m^-1^) | 59 | 61 | 70 | 48 | 81 |
| pH (-) | 8 ± 1 | 8 ± 1 | 8 ± 1 | 8 ± 1 | 8 ± 1 |
| Dissolved oxygen concentration (mg L^-1^) | 8.6 ± 0.5 | 8.4 ± 0.6 | 8.5 ± 0.4 | 8.7 ± 0.4 | 9.2 |

Table S7. Partitioning of MFN, MET and TER between the sediment and the water phases in individual (ONE) and multi-contaminated (MIX) experiments under biotic and abiotic conditions.


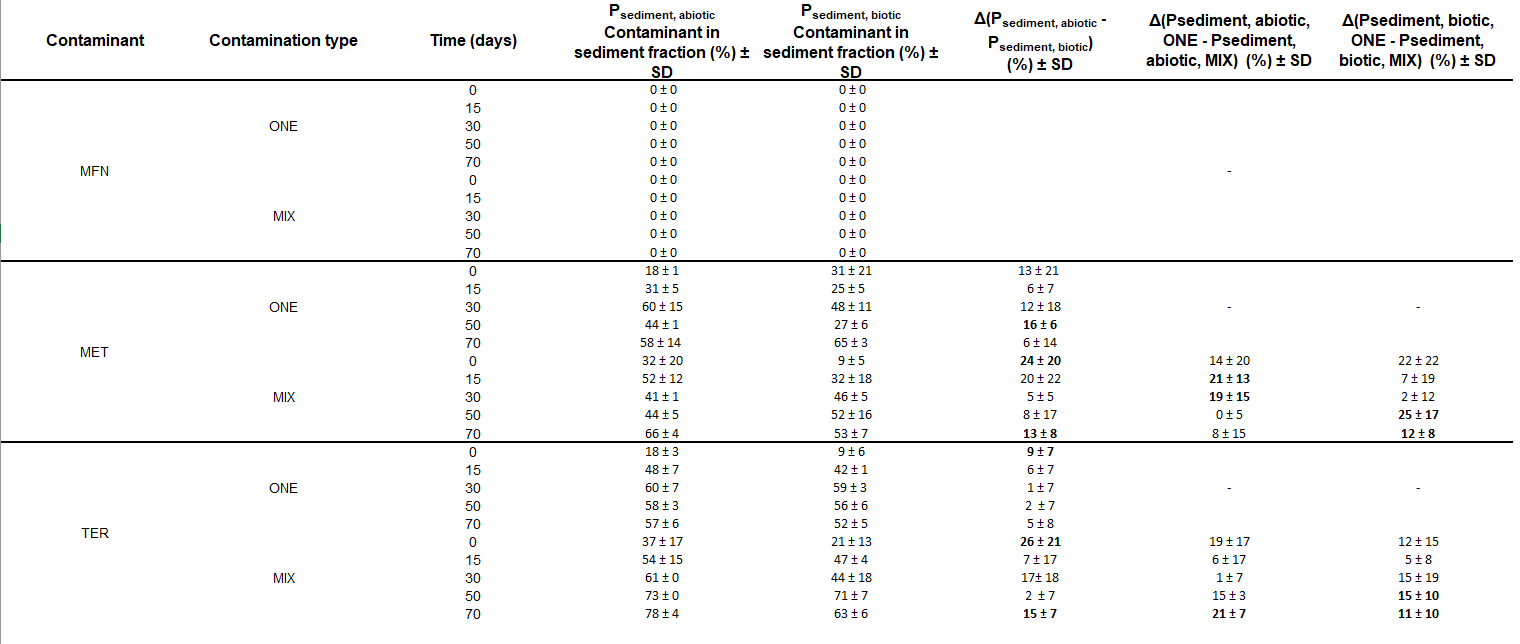


***P_sediment,t_*:** micropollutant concentration at time t in the sediment compartment (sediment fraction). ***Pwater t*:** micropollutant concentration at time t in the water compartment (dissolved fraction)

***SD***: standard deviation was estimated based on the equation $SD(C)=\sqrt{\left( \mathrm{SD}\left( A \right)^{2}+SD\left( B \right)^{2} \right)}$

***Δ(P)_biot_:*** difference in partitioning of biotic and abiotic experiments

***Δ(P)_mix_:*** difference in partitioning of SIN and MIX experiments under biotic (***Δ(P)mix, biotic)*** and a biotic (***Δ(P)mix, abiotic)*** conditions

|  | Bacterial NPMANOVA | | | | | |
| --- | --- | --- | --- | --- | --- | --- |
|  | Factors | Degree of freedom | Sum of Squares | R² | F-statistic | p-value |
|  | Time (1) | 1 | 1.8525 | 0.13323 | 15.7432 | 0.0001 |
|  | Matrix (2) | 1 | 1.8307 | 0.13166 | 15.5574 | 0.0001 |
|  | Contamination (3) | 4 | 1.6538 | 0.11894 | 3.5136 | 0.0001 |
| I**nteractions** | (1) : (2) | 1 | 0.6107 | 0.04392 | 5.1898 | 0.0001 |
|  | (1) : (3) | 4 | 1.2585 | 0.09051 | 2.6737 | 0.0002 |
|  | (2) : (3) | 4 | 1.1737 | 0.08441 | 2.4935 | 0.0002 |
|  | (1) : (2) : (3) | 4 | 0.9354 | 0.06728 | 1.9874 | 0.0008 |
|  | Residual | 39 | 4.5892 | 0.3305 |  |  |
|  | Total | 58 | 13.9045 | 1 |  |  |

Table S8. Non-parametric multivariate analysis of variance (NPMANOVA) of bacterial communities in water-sediment microcosms account for the main experimental factors. Time, Matrix, Contamination (i.e., CTRL, MFN, MET, TER, and MIX)

Table S9. Non-parametric multivariate analysis of variance (NPMANOVA) of archaeal communities in water-sediment microcosms account for the main experimental factors. Time, Matrix, Contamination (i.e., CTRL, MFN, MET, TER, and MIX)

|  | Archaeal NPMANOVA | | | | | |
| --- | --- | --- | --- | --- | --- | --- |
|  | Factors | Degree of freedom | Sum of Squares | R² | F-statistic | p-value |
|  | Time (1) | 1 | 2.5425 | 0.18950 | 20.8283 | 0.0001 |
|  | Matrix (2) | 1 | 1.3213 | 0.09849 | 10.8246 | 0.0001 |
|  | Contamination (3) | 4 | 1.1375 | 0.08478 | 2.3296 | 0.0003 |
| I**nteractions** | (1) : (2) | 1 | 0.5256 | 0.03917 | 4.3057 | 0.0001 |
|  | (1) : (3) | 4 | 1.0289 | 0.07669 | 2.1072 | 0.0012 |
|  | (2) : (3) | 4 | 1.1461 | 0.08543 | 2.3473 | 0.0005 |
|  | (1) : (2) : (3) | 4 | 0.9540 | 0.07110 | 1.9537 | 0.0042 |
|  | Residual | 39 | 4.7607 | 0.35484 |  |  |
|  | Total | 58 | 13.4166 | 1 |  |  |

Table S10. Relative abundance (%) of phyla in sediment compared to water phases with statistical significance determined by Wilcoxon tests. n.s: non-significant.

| **Phylum** | **Sediment (mean ± SD%)** | **Water (mean ± SD%)** | **Significance (p≤0.05)** |
| --- | --- | --- | --- |
| Unassigned | 0.01 ± 0.03 | 0.14 ± 0.5 | n.s |
| Unassigned Archaea | 0 ± 0.02 | 0 ± 0.01 | n.s |
| Unassigned Bacteria | 0.65 ± 0.29 | 0.36 ± 0.25 | * |
| Acetothermia | 0 ± 0.01 | 0 ± 0.01 | n.s |
| Acidobacteriota | 4.28 ± 1.13 | 1.77 ± 1.64 | * |
| Actinobacteriota | 12.86 ± 3.46 | 5.27 ± 3.07 | * |
| Armatimonadota | 0.06 ± 0.05 | 0.02 ± 0.02 | * |
| Bacteroidota | 9.24 ± 3.56 | 8.2 ± 6.81 | * |
| Bdellovibrionota | 0.17 ± 0.19 | 0.8 ± 3.21 | n.s |
| Caldisericota | 0.01 ± 0.02 | 0.01 ± 0.01 | n.s |
| Campilobacterota | 0.48 ± 0.6 | 1.27 ± 1.69 |  |
| Chloroflexi | 7.91 ± 2.66 | 2.46 ± 1.81 | * |
| Crenarchaeota | 2.03 ± 1.45 | 1.21 ± 1.15 | * |
| Cyanobacteria | 0.18 ± 0.33 | 0.2 ± 0.46 | n.s |
| DTB120 | 0.13 ± 0.15 | 0.03 ± 0.04 | * |
| Deferrisomatota | 0.02 ± 0.03 | 0.01 ± 0.02 | n.s |
| Dependentiae | 0.02 ± 0.05 | 0.02 ± 0.05 | n.s |
| Desulfobacterota | 7.62 ± 2.46 | 3.5 ± 2.28 | * |
| Elusimicrobiota | 0.04 ± 0.04 | 0.07 ± 0.09 | n.s |
| Euryarchaeota | 0.1 ± 0.14 | 0.02 ± 0.03 | * |
| Fibrobacterota | 0.03 ± 0.02 | 0.01 ± 0.03 | n.s |
| Firmicutes | 7.34 ± 2.01 | 5.65 ± 5.42 | * |
| Gemmatimonadota | 1.55 ± 0.44 | 0.9 ± 0.54 | * |
| Halobacterota | 3.25 ± 4.1 | 1.45 ± 2.56 | * |
| Hydrogenedentes | 0 ± 0.01 | 0 ± 0.01 | n.s |
| Iainarchaeota | 0 ± 0 | 0.06 ± 0.19 | * |
| LCP-89 | 0.02 ± 0.03 | 0.01 ± 0.01 | * |
| Latescibacterota | 0.21 ± 0.11 | 0.07 ± 0.07 | * |
| MBNT15 | 0.32 ± 0.17 | 0.09 ± 0.09 | * |
| Methylomirabilota | 0.08 ± 0.07 | 0.02 ± 0.02 | * |
| Micrarchaeota | 0 ± 0 | 0.02 ± 0.08 | * |
| Myxococcota | 1.46 ± 0.81 | 0.55 ± 0.37 | * |
| NB1-j | 0.77 ± 0.27 | 0.33 ± 0.23 | * |
| Nanoarchaeota | 0.12 ± 0.14 | 1.07 ± 1.92 | * |
| Nitrospinota | 0.07 ± 0.07 | 0 ± 0.02 | * |
| Nitrospirota | 2.27 ± 0.82 | 1.34 ± 0.88 | * |
| Patescibacteria | 0.35 ± 0.75 | 1.24 ± 3.12 | * |
| Planctomycetota | 0.01 ± 0.01 | 0.11 ± 0.22 | * |
| Proteobacteria | 34.4 ± 5.46 | 59.92 ± 17.18 | * |
| SAR324_clade (Marine_group_B) | 0.01 ± 0.02 | 0.03 ± 0.12 | n.s |
| Spirochaetota | 0.37 ± 0.35 | 0.19 ± 0.23 | * |
| Sva0485 | 0.09 ± 0.11 | 0.02 ± 0.03 | * |
| TA06 | 0.01 ± 0.02 | 0 ± 0 | * |
| Thermoplasmatota | 0.02 ± 0.03 | 0.01 ± 0.03 | n.s |
| Verrucomicrobiota | 1.33 ± 0.74 | 1.51 ± 1.11 | * |
| WOR-1 | 0 ± 0 | 0 ± 0.01 | n.s |
| WPS-2 | 0.02 ± 0.05 | 0.04 ± 0.12 | n.s |
| WS4 | 0 ± 0.02 | 0 ± 0.01 | n.s |
| Zixibacteria | 0.19 ± 0.14 | 0.06 ± 0.07 | * |

Table S11. Relative abundance (%) of phyla on day 70 compared to day 0, including both sediment and water phases, with statistical significance determined by the Wilcoxon test. n.s: non-significant.

| **Phylum** | **Day 70 (mean ± SD%)** | **Day 0 (mean ± SD%)** | **Significance (p≤0.05)** |
| --- | --- | --- | --- |
| Unassigned | 0.13 ± 0.5 | 0.01 ± 0.03 | n.s |
| Unassigned Archaea | 0.01 ± 0.02 | 0 ± 0 | n.s |
| Unassigned Bacteria | 0.62 ± 0.37 | 0.39 ± 0.14 | * |
| Acetothermia | 0.01 ± 0.02 | 0 ± 0 | n.s |
| Acidobacteriota | 2.29 ± 1.54 | 3.74 ± 1.95 | * |
| Actinobacteriota | 8.02 ± 4.52 | 10.01 ± 5.37 | * |
| Armatimonadota | 0.05 ± 0.06 | 0.03 ± 0.02 | * |
| Bacteroidota | 6.74 ± 6.5 | 10.76 ± 3.01 | * |
| Bdellovibrionota | 0.84 ± 3.21 | 0.13 ± 0.18 | n.s |
| Caldisericota | 0 ± 0.01 | 0.02 ± 0.02 | n.s |
| Campilobacterota | 0.14 ± 0.15 | 1.65 ± 1.56 | n.s |
| Chloroflexi | 5.85 ± 4.28 | 4.4 ± 2.45 | * |
| Crenarchaeota | 2.02 ± 1.38 | 1.19 ± 1.21 | * |
| Cyanobacteria | 0.32 ± 0.52 | 0.06 ± 0.07 | n.s |
| DTB120 | 0.12 ± 0.15 | 0.04 ± 0.03 | * |
| Deferrisomatota | 0.02 ± 0.03 | 0.02 ± 0.02 | n.s |
| Dependentiae | 0.03 ± 0.07 | 0 ± 0.01 | n.s |
| Desulfobacterota | 5.41 ± 4.03 | 5.64 ± 1.87 | * |
| Elusimicrobiota | 0 ± 0.01 | 0.11 ± 0.07 | n.s |
| Euryarchaeota | 0.11 ± 0.13 | 0.01 ± 0.04 | * |
| Fibrobacterota | 0.02 ± 0.02 | 0.02 ± 0.03 | n.s |
| Firmicutes | 7.5 ± 5.38 | 5.42 ± 1.91 | * |
| Gemmatimonadota | 1.23 ± 0.69 | 1.22 ± 0.48 | * |
| Halobacterota | 3.51 ± 4.4 | 1.12 ± 1.5 | * |
| Hydrogenedentes | 0 ± 0 | 0 ± 0.01 | n.s |
| Iainarchaeota | 0.06 ± 0.19 | 0 ± 0 | * |
| LCP-89 | 0.02 ± 0.03 | 0 ± 0.01 | * |
| Latescibacterota | 0.16 ± 0.13 | 0.12 ± 0.1 | * |
| MBNT15 | 0.25 ± 0.22 | 0.15 ± 0.1 | * |
| Methylomirabilota | 0.06 ± 0.07 | 0.04 ± 0.04 | * |
| Micrarchaeota | 0.02 ± 0.08 | 0 ± 0 | * |
| Myxococcota | 1.16 ± 0.97 | 0.82 ± 0.44 | * |
| NB1-j | 0.62 ± 0.4 | 0.47 ± 0.22 | * |
| Nanoarchaeota | 1.15 ± 1.87 | 0.03 ± 0.04 | * |
| Nitrospinota | 0.02 ± 0.04 | 0.05 ± 0.07 | * |
| Nitrospirota | 2 ± 1.16 | 1.58 ± 0.66 | * |
| Patescibacteria | 1.35 ± 3.16 | 0.24 ± 0.27 | * |
| Planctomycetota | 0.03 ± 0.09 | 0.09 ± 0.22 | * |
| Proteobacteria | 46.2 ± 21.44 | 48.59 ± 14.12 | * |
| SAR324_clade (Marine_group_B) | 0.01 ± 0.02 | 0.04 ± 0.12 | n.s |
| Spirochaetota | 0.37 ± 0.39 | 0.18 ± 0.15 | * |
| Sva0485 | 0.1 ± 0.1 | 0.01 ± 0.01 | * |
| TA06 | 0.01 ± 0.02 | 0 ± 0 | * |
| Thermoplasmatota | 0.03 ± 0.04 | 0.01 ± 0.02 | n.s |
| Verrucomicrobiota | 1.19 ± 0.76 | 1.66 ± 1.07 | * |
| WOR-1 | 0 ± 0.01 | 0 ± 0.01 | n.s |
| WPS-2 | 0.05 ± 0.12 | 0 ± 0 | n.s |
| WS4 | 0.01 ± 0.02 | 0 ± 0 | n.s |
| Zixibacteria | 0.18 ± 0.16 | 0.07 ± 0.05 | * |

|  |  |  |  | | | | |
| --- | --- | --- | --- | --- | --- | --- | --- |
|  |  | *Dataset* | *Total dataset* | *Sediment d0* | *Water d0* | *Sediment d70* | *Water d70* |
|  |  | *Confounding factors* | *matrix:time* | *-* | *-* | *-* | *-* |
| **Tested hypotheses** |  | CTRL : MFN | **0.019** | 0.100 | 0.100 | 0.100 | 0.375 |
|  |  | CTRL : MET | **0.001** | 0.100 | 0.100 | 0.100 | 0.333 |
|  |  | CTRL : TER | **0.001** | 0.100 | 0.100 | 0.100 | 0.333 |
|  |  | CTRL : MIX | **0.001** | 0.100 | 0.100 | 0.100 | 0.250 |
|  |  | MFN : MET | **0.001** | 0.100 | 0.100 | 0.100 | 0.444 |
|  |  | MFN : TER | **0.008** | 0.100 | 0.100 | 0.100 | 0.375 |
|  |  | MFN : MIX | **0.001** | 0.100 | 0.100 | 0.100 | 0.250 |
|  |  | MET : TER | **0.008** | 0.100 | 0.100 | 0.100 | 0.500 |
|  |  | MET : MIX | **0.001** | 0.100 | 0.100 | 0.100 | 0.250 |
|  |  | TER : MIX | **0.001** | 0.100 | 0.100 | 0.100 | 0.250 |
|  |  | Samples in dataset (n) | 59 | 15 | 14 | 15 | 15 |
|  |  | Samples per hypothesis (h) | 24 or 23* | 6 | 6 or 5* | 6 | 6 |
|  |  | Samples per factor (f) | 12 or 11* | 3 | 3 or 2* | 3 | 3 |

Table S12. Non-parametric multivariate analysis of variance (NPMANOVA) for bacterial communities in water-sediment microcosms. Pairwise comparisons were performed to assess differences in prokaryotic communities. Analysis included all combinations of contamination types such as CTRL, MFN, MET, TER, and MIX. Benjamini-Hochberg correction was applied to account for multiple comparisons. Permutations were constrained based on confounding factors matrix and time for the total dataset. Analyses were conducted without constraining permutations for four distinct subgroups: sediment and water samples collected at days 0 and day 70.

Table S13. Non-Parametric Multivariate Analysis of Variance (NPMANOVA) for bacterial Communities in Water-Sediment Microcosms. Pairwise comparisons were performed to assess differences in prokaryotic communities within water-sediment microcosms. The analysis included an aggregated subgroup of contamination types ONE referring to the pool of samples under individual contamination (with a sized-up sample size, combining MFN, MET, and TER samples), then the MIX and the CTRL samples. Benjamini-Hochberg correction was applied to account for multiple comparisons. Permutations were constrained based on confounding factors, specifically matrix and time, for the entire dataset (total dataset). Additionally, analyses were conducted without constraining permutations for four distinct subgroups: sediment samples collected at days 0 (sediment d0) or day 70 (sediment d70) and water samples collected at days 0 (water d0) or day 70 (water d70).

|  |  |  |  | Pairwise NPMANOVA p-values, using Benjamini-Hochberg correction for multiplicity | | | | | |  |
| --- | --- | --- | --- | --- | --- | --- | --- | --- | --- | --- |
|  |  | *Dataset* | | | *Total dataset* | *Sediment d0* | *Water d0* | *Sediment d70* | *Water d70* | |
|  |  | *Confounding factors* | | | *matrix:time* | *-* | *-* | *-* | *-* | |
| **Tested hypothsessis** |  | CTRL : MIX | | | **0.002** | 0.100 | 0.100 | 0.100 | 0.150 | |
|  |  | CTRL : ONE (**size up**) | | | **0.002** | **0.009** | **0.042** | **0.006** | 0.208 | |
|  |  | MIX : ONE (**size up**) | | | **0.002** | **0.009** | **0.042** | **0.006** | **0.039** | |
|  |  | Samples in dataset (n) | | | 59 | 15 | 14 | 15 | 15 | |
|  |  | Samples per hypothesis (h) | | | 24 or ***47*** ***(ONE)*** | 6 or ***12***  ***(ONE)*** | 6 or ***11* *(ONE)*** | 6 or ***12***  ***(ONE)*** | 6 or ***12 (ONE)*** | |
|  |  | Samples per factor (f) | | | 12 or ***36***  ***(ONE)*** | 3 or ***9***  ***(ONE)*** | 3 or ***8***  ***(ONE)*** | 3 or ***9***  ***(ONE)*** | 3 or ***9***  ***(ONE)*** | |

Table S14. Non-parametric multivariate analysis of variance (NPMANOVA) for archaeal communities in water-sediment microcosms. Pairwise comparisons were performed to assess differences in prokaryotic communities. Analysis included all combinations of contamination types such as CTRL, MFN, MET, TER, and MIX. Benjamini-Hochberg correction was applied to account for multiple comparisons. Permutations were constrained based on confounding factors matrix and time for the total dataset. Analyses were conducted without constraining permutations for four distinct subgroups: sediment and water samples collected at days 0 and day 70.

|  |  |  |  | | | | |
| --- | --- | --- | --- | --- | --- | --- | --- |
|  |  | *Dataset* | *Total dataset* | *Sediment d0* | *Water d0* | *Sediment d70* | *Water d70* |
|  |  | *Confounding factors* | *matrix:time* | *-* | *-* | *-* | *-* |
| **Tested hypotheses** |  | CTRL : MFN | 0.102 | 0.100 | 0.100 | 0.100 | 0.375 |
|  |  | CTRL : MET | 0.081 | 0.100 | 0.100 | 0.100 | 0.333 |
|  |  | CTRL : TER | **0.043** | 0.100 | 0.100 | 0.100 | 0.333 |
|  |  | CTRL : MIX | 0.109 | 0.100 | 0.100 | 0.100 | 0.250 |
|  |  | MFN : MET | **0.011** | 0.100 | 0.100 | 0.100 | 0.444 |
|  |  | MFN : TER | **0.008** | 0.100 | 0.100 | 0.100 | 0.375 |
|  |  | MFN : MIX | **0.002** | 0.100 | 0.100 | 0.100 | 0.250 |
|  |  | MET : TER | 0.341 | 0.100 | 0.100 | 0.100 | 0.500 |
|  |  | MET : MIX | **0.012** | 0.100 | 0.100 | 0.100 | 0.250 |
|  |  | TER : MIX | **0.004** | 0.100 | 0.100 | 0.100 | 0.250 |
|  |  | Samples in dataset (n) | 59 | 15 | 14 | 15 | 15 |
|  |  | Samples per hypothesis (h) | 24 or 23* | 6 | 6 or 5* | 6 | 6 |
|  |  | Samples per factor (f) | 12 or 11* | 3 | 3 or 2* | 3 | 3 |

Table S15. Proportion of fold changes (FC) observed from CTRL to MFN, MET, TER, and MIX contamination types at different taxonomic levels. Fold changes with an exact value of 1, indicating the absence of taxa in both contaminant types and CTRL experiments, were excluded from the analysis. Proportions were adjusted by eliminating absent taxa.

|  | Proportion (%)  FC>1 | Proportion (%)  FC<1 |
| --- | --- | --- |
| Kingdom | 38 | 63 |
| Phylum | 63 | 37 |
| Class | 63 | 37 |
| Order | 65 | 35 |
| Family | 65 | 35 |
| Genus | 63 | 38 |
| Species | 62 | 38 |
| ASVs | 61 | 39 |

1. Supplementary Methods

Method S1

**MET and TER quantification with GCMS**

MET and TER were quantified with a gas chromatograph (GC, Trace 1300, ThermoFisher Scientific) coupled to a mass spectrometer (MS, ISQ™, ThermoFisher Scientific). The chromatographic separation was performed with a TG-5MS column (30 m × 0.25 mm ID, 0.25 µm film thickness)^5^. Samples were injected at a temperature of 280°C in split less mode with a carrier flow of 1.5 mL/min. Column was heated at a temperature of 50°C for 2 min and then heat up to 150°C at a rate of 30°C/min. Temperature was increased to 180°C at a rate of 2°C/min, then to 280°C at a rate of 15°C/min, held for 1 min, further increased to 330°C at a rate of 30°C/min and held at 330°C for 1 min.

**Quantification of micropollutants and transformation products with LCMS**

MFN and its transformation products (TPs) were quantified using an Ultra High Performance Liquid Chromatography (UHPLC, Ultimate 3000, Thermo Fisher Scientific) coupled with a triple quadrupole mass spectrometer (MS/MS, TSQ Quantiva, Thermo Fisher Scientific) equipped with a Accucore aQ C18 column (100 x 2.1 mm, 2.6 µm granulometry, Thermo Fischer Scientific). The column and autosampler temperatures were 20°C and 9°C, respectively. The sample (10 µL) containing metformin-d6 (at 200 µg L^-1^) as an internal standard was injected with an ACC-3000 autosampler (Ultimate 3000, Thermo Fisher Scientific). LC-grade water and acetonitrile acidified with 0.1% and 0.05% formic acid respectively were used as eluents for the chromatographic gradient at a flow rate of 0.3 mL/min (10% to 40% acetonitrile in 1 min, 40 to 90% acetonitrile in 20 s, isocratic elution at 90% acetonitrile for 40s, 90% to 10% acetonitrile in 15 s, and reconditioning in 10% acetonitrile for 30 s). The MS/MS was operated at an ionization voltage of 1500V (in positive mode) and 2000 V (in negative mode), CID gas at 1.5 mTorr and vaporizing temperature of 300 °C. Precursors and fragments ions were acquired in multi reaction mode (MRM).

The protocol was adapted for MET and TER transformation products. Briefly, for MET, LC-grade water and acetonitrile acidified with 0.1% and 0.05% formic acid respectively were used as eluents for the chromatographic gradient at a flow rate of 0.3 mL/min. The program involved a gradient of 10% to 90% acetonitrile in 8 min followed by isocratic elution at 90% for 1 min, 90% to 10% acetonitrile in 1 min, and reconditioning in 10% acetonitrile for 3 min. For TER, LC-grade water and methanol acidified with 0.1% and 0.05% formic acid were used as eluents for the chromatographic gradient at a flow rate of 0.4 mL/min. The program involved a gradient of 10% to 90% acetonitrile in 8 min, followed by isocratic elution at 90% for 5 min, 90% to 10% acetonitrile in 1 min and reconditioning at 10% acetonitrile for 3 min. In this case, the MS/MS was operated at an ionization voltage of 1800 V (in positive mode), CID gas at 1.5 mTorr and vaporizing temperature of 300 °C.

1. Supplementary Figures


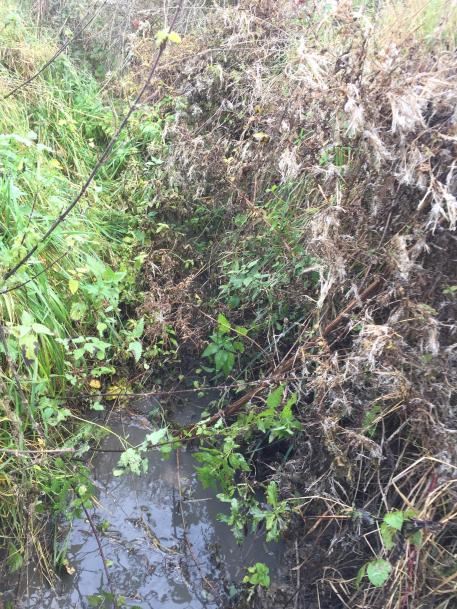

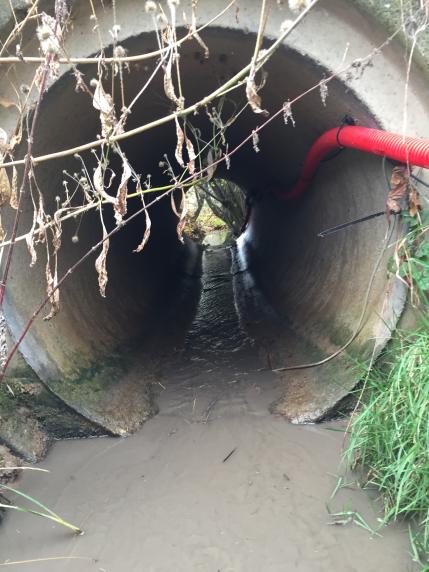


Figure S1. Avenheimerbach riverbed (France, 48°39’58.08” N, 07°35’36.92” E), sampling area.


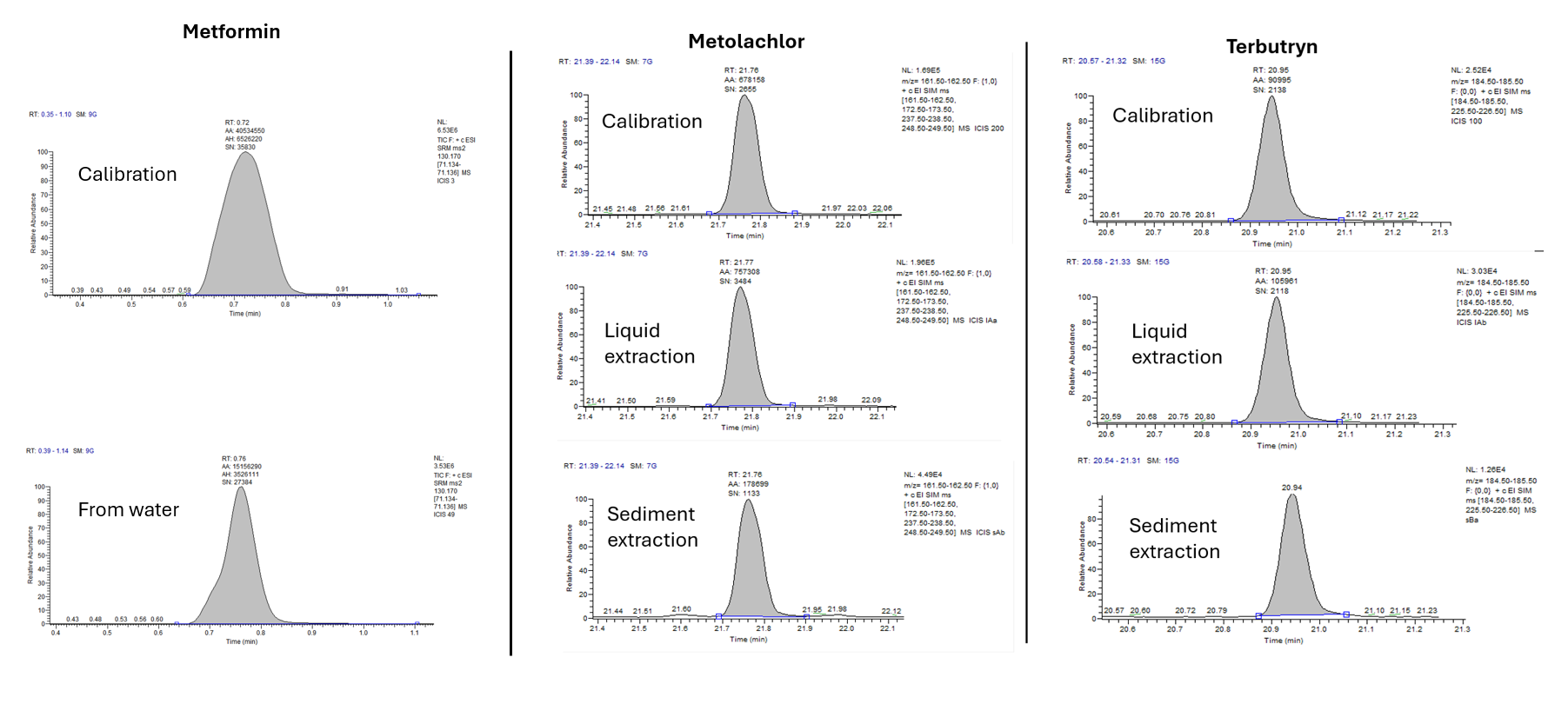


Figure S2. Absence of matrix effect for MFN (LC/MSMS) after sampling from water, and MET (GC/MS) TER(GC/MS) after water and sediment extraction.


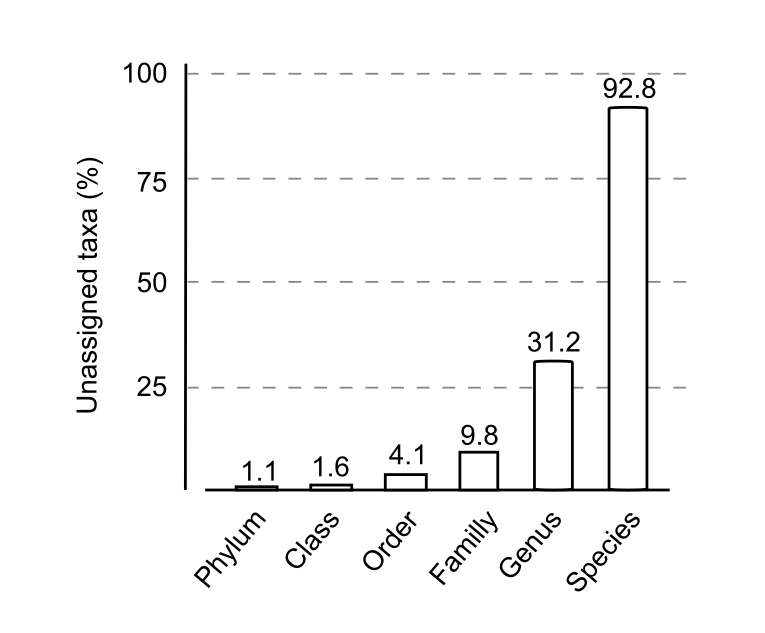


Figure S3. Number of unassigned taxa for each taxonomic level.


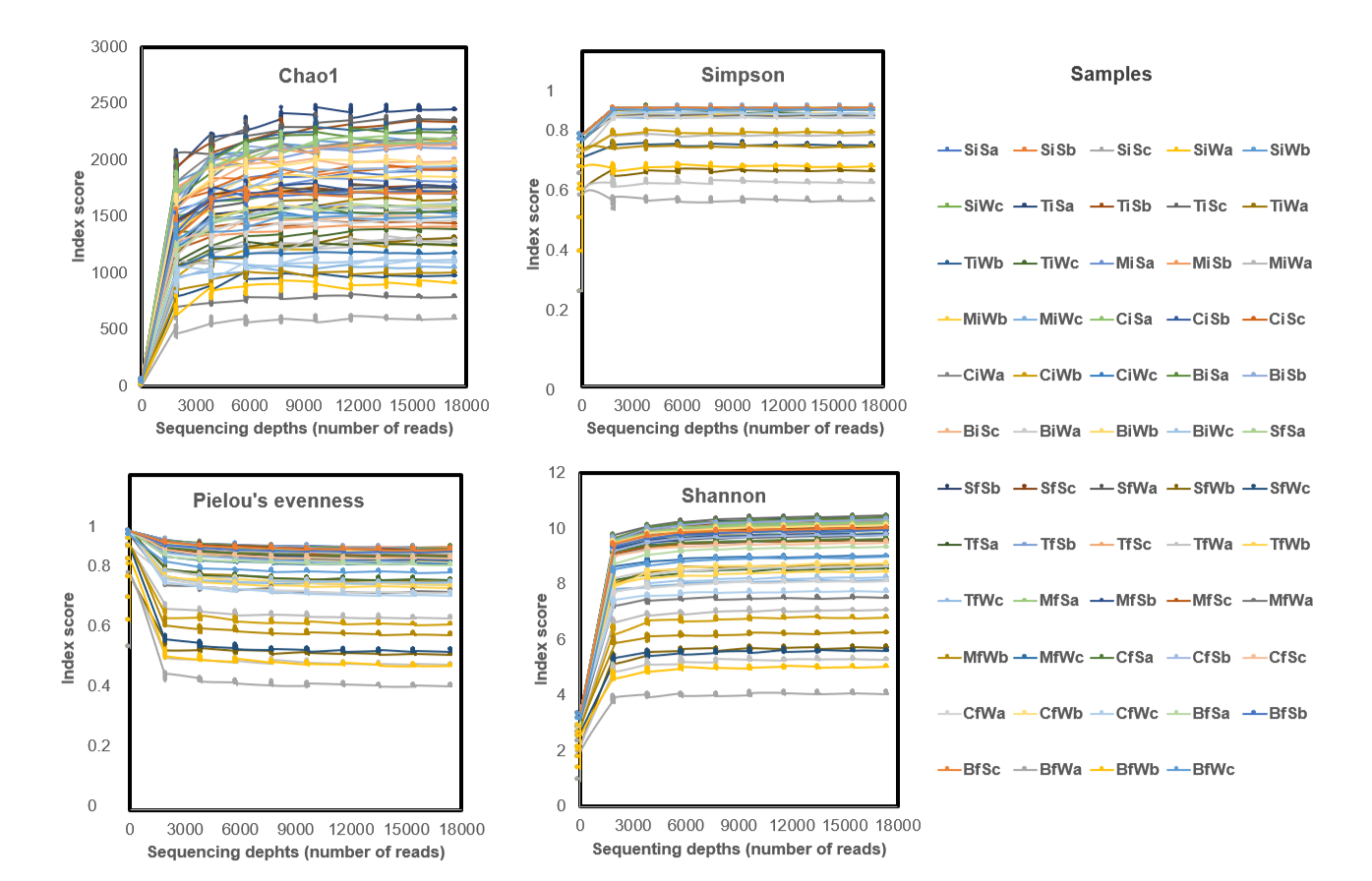


Figure S4. Samples rarefaction curves for Chao1, Simpson, Pielou’s evenness, and Shannon indices. Indices were obtained at 10, 1946, 3883, 5820, 7756, 9693, 11630, 13566, 15503, and 17440 reads.

Figure S5. Number of reads obtained for each water and sediment sample.


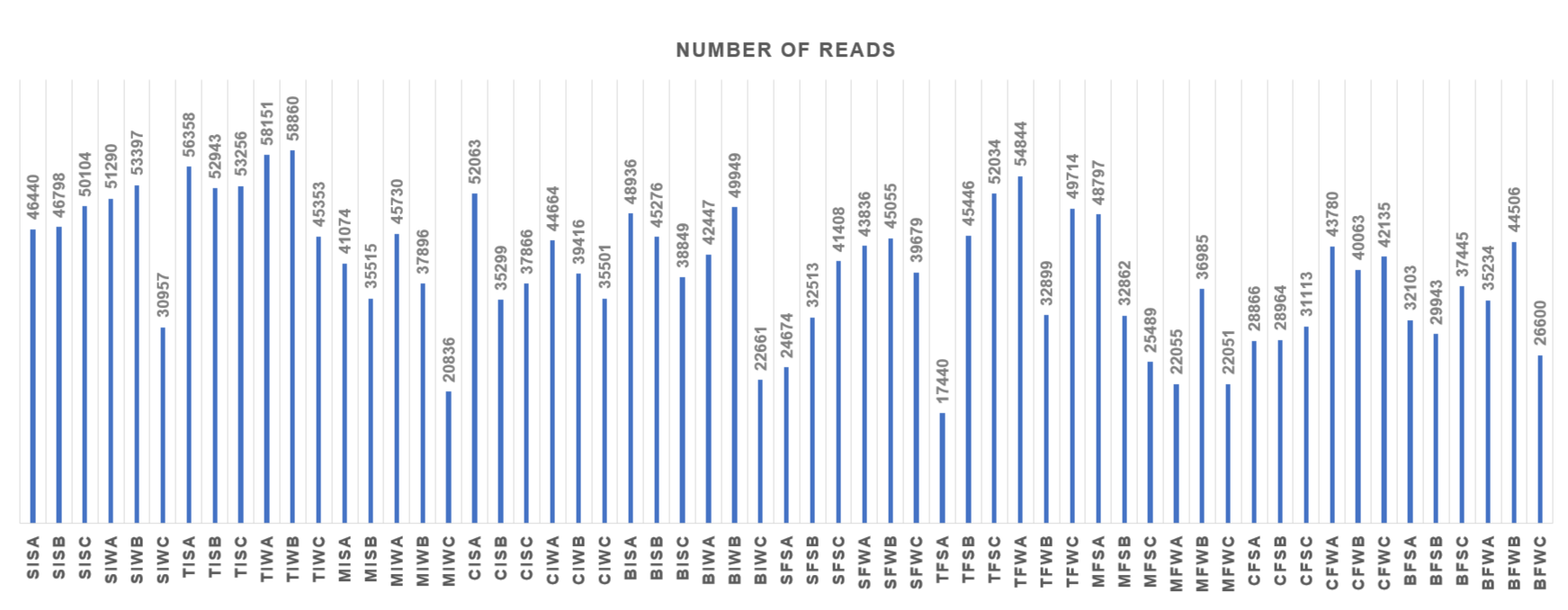


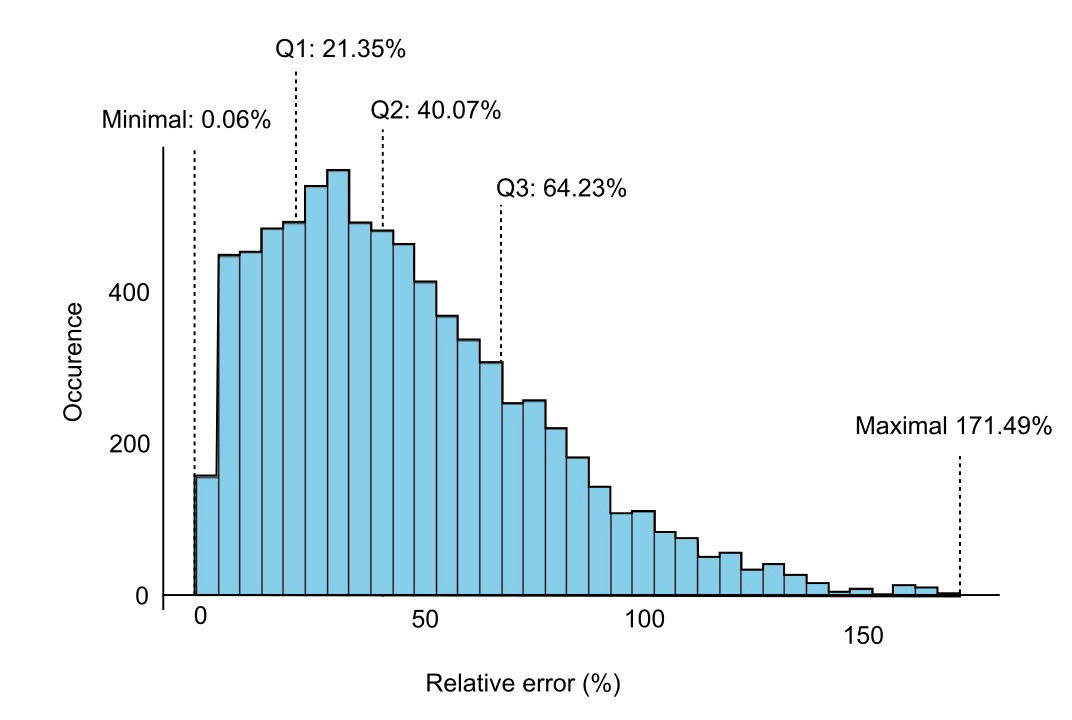


Figure S6. Distribution of relative error between triplicates for all ASVs abundance.


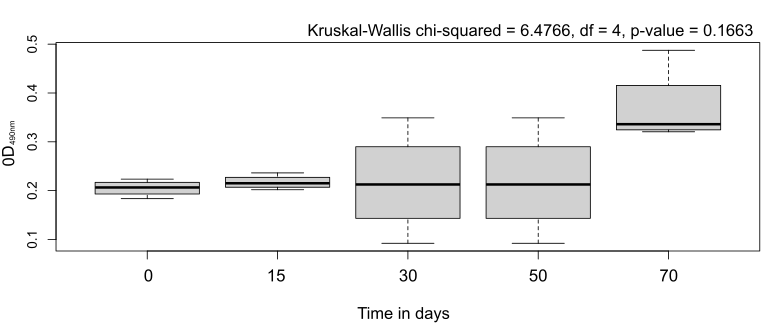


Figure S7. Microbial activity from day 0 to day 70 (FDA).


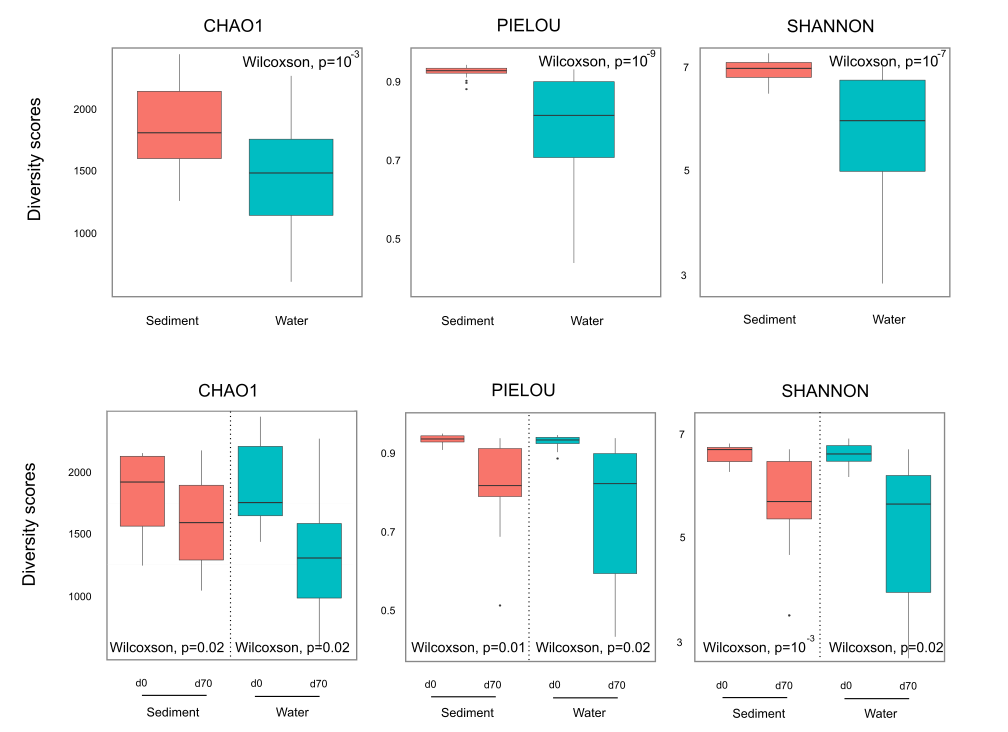


Figure S8. Variations in alpha-diversity from sediment to water and d0 to d70 in each phase. Results of Wilcoxon tests were presented in the caption.
